# The Impact of Dietary Encapsulated Fennel Seed (*Foeniculum vulgare* Mill.) Essential Oil Inclusion Levels on Performance, Serum Hormone Profiles, and Expression of Reproductive Axis‐Related Genes in the Early and Late Laying Phases of Hens

**DOI:** 10.1002/vms3.70150

**Published:** 2024-12-10

**Authors:** Hasan Hüseyin İpçak, Muzaffer Denli, Beran Yokuş, Servet Bademkıran

**Affiliations:** ^1^ Department of Animal Science, Faculty of Agriculture Dicle University Diyarbakır Turkey; ^2^ Department of Biochemistry, Faculty of Veterinary Medicine Dicle University Diyarbakır Turkey; ^3^ Department of Obstetrics and Gynaecology, Faculty of Veterinary Medicine Dicle University Diyarbakir Turkey

**Keywords:** fennel oil, gene expression, hormone, laying hen, performance, reproductive

## Abstract

Fennel seed (*Foeniculum vulgare* Mill.) essential oil (FEO), which is rich in the phytoestrogenic compound trans‐anethole, interacts with oestrogen receptors and influences molecular targets within cells and hormonal responses. This study examined the effect of dietary encapsulated FEO inclusion levels on performance, reproductive hormone profiles, and gene expression in laying hens during the early and late phases. The study was conducted in two independent trials, each involving 210 Atak‐S laying hens that were randomly distributed into 3 experimental groups, each having 10 replicates with 7 hens. The dietary treatments included a basal diet without FEO (Control) and a basal diet supplemented with 175 (FEO175) or 350 mg (FEO350) of encapsulated FEO/kg for 12 weeks. The results showed that FEO350 treatment improved egg production, egg mass, and feed conversion ratio during both early and late phases (*p* < 0.05). Moreover, increasing FEO inclusion levels enhanced oestradiol, follicle‐stimulating hormone, luteinizing hormone and progesterone concentrations in both early and late laying hens, reaching peak levels at FEO350 (*p* < 0.05). FEO supplementation upregulated the expression of oestrogen receptor 2 (ESR2) and follicle‐stimulating hormone receptor (FSHR) (*p* < 0.05). Furthermore, FEO350 increased prolactin receptor (PRLR) expression during the early phase but decreased it during the late laying phase (*p* < 0.05). Positive correlations were observed between egg production and FSHR, ESR2 and steroidogenic acute regulatory protein (STAR) expression, with a negative correlation for PRLR (*p* < 0.05). In conclusion, 350 mg FEO/kg was found to be the most effective level for enhancing layer performance.

## Graphical abstract

Encapsulated fennel seed essential oil at 350 mg/kg improved egg production and laying performance by enhancing oestradiol, FSH, LH and progesterone levels and upregulating related reproductive genes in laying hens during the early and late phases.

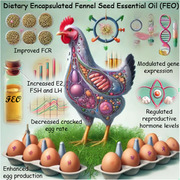

## 1 Introduction

Egg production in poultry is a multifaceted process influenced by genetics, endocrine system, immunity, nutrition, environmental factors and management practices (Liu et al. 2018). This process is strictly dependent on the growth and maturation of different follicles, which follows a hierarchical sequence from pre‐hierarchical to hierarchical in the ovary (Asiamah et al. 2021). Unlike mammals, poultry exhibits a unique ovulatory cycle characterized by the absence of a corpus luteum and a luteal phase. This cycle revolves around the follicular phase and ovulation, ensuring a continuous cycle conducive to high egg production in laying hens (Johnson 2014). However, the egg production cycle in laying hens is tightly controlled by a cascade of hormonal signals that regulate proper growth and maturation of ovarian follicles, ovulation, eggshell formation and egg laying. The hormonal mechanism of egg formation in hens is a finely tuned process that involves the interplay of several hormones, including gonadotropin‐releasing hormone (GnRH), follicle‐stimulating hormone (FSH), luteinizing hormone (LH), oestrogen, progesterone, prolactin and testosterone, which are primarily regulated by the hypothalamic‐pituitary‐gonadal (HPG) axis (Zhang et al. 2022). An intricate hormonal balance governing the egg‐laying cycle is essential for achieving high egg productivity. Hormonal imbalances or age‐related changes can diminish or halt egg production in laying hens. Consequently, understanding these complex hormonal mechanisms is imperative for managing reproductive health and optimizing egg production in poultry farms. In commercial poultry farming, indirect control over the hormonal balance of laying hen is typically achieved through lighting programmes, nutritional strategies and general health management practices. Regulation of the ovulatory cycle in hens via hormonal and molecular pathways underscores the pivotal role of dietary components in enhancing productivity and laying performance. Furthermore, examining physiological changes and their impacts along with nutritional regimes is crucial for identifying productivity‐enhancing practices in poultry farming. *Foeniculum vulgare* Mill, commonly known as fennel, has emerged as a valuable source of nutrition for both animals and humans, offering significant benefits to the digestive, endocrine, reproductive and respiratory systems (Badgujar, Patel, and Bandivdekar 2014). Abou‐Elkhair, Selim, and Hussein (2018) demonstrated that dietary supplementation with fennel seeds improved egg weight, egg production, egg mass and feed conversion ratio (FCR) in laying hens. Gharaghani, Shariatmadari, and Torshizi (2015) observed that fennel fruit intake, owing to its antioxidant properties, could alleviate the adverse effects of free radicals on laying hens under heat stress conditions. In addition, Haglan and Majed (2022) reported an improvement in egg quality when fennel seeds were used. Supplementing laying hen diets with different sources of anethole has been found to improve eggshell quality (Beshara 2018). The compiled data indicated that fennel has multiple beneficial effects on hen health and egg production. However, there is limited information regarding the effectiveness of fennel in terms of reproductive and physiological efficiency in laying hens.

Fennel contains active compounds with oestrogen‐like effects, notably anethole, a phytoestrogen that mimics the action of oestrogen in animals and interacts with oestrogen receptors (ER) (Christaki et al. 2011; Wang et al. 2022). Oestrogen plays a central role in regulating the reproductive system by affecting the ovulatory cycle, oviduct development, follicle maturation and egg laying (Johnson 2014). The effects of oestrogen on target cells are mediated through ER, found inside and on the cell surface (Griffin et al. 1999; Johnson 2015), expressed not only in the hypothalamus and pituitary gland but also in the ovary and oviduct of hens. Activated by oestrogen, ERs are transported to the nucleus, bind to DNA, and regulate gene expression (Griffin et al. 1999). Ghasemian et al. (2020) reported that fennel essential oil is rich in anethole, which interacts with molecular targets. Consequently, fennel seed essential oil (FEO) and its anethole contents may influence the reproductive physiological efficiency of commercial laying hens through mechanisms involving molecular and hormonal pathways. However, despite familiarity with hormonal functions in the ovulatory cycle of hens, detailed studies are lacking on how fennel affects hormones at each ovulatory stage, regulates gene expression associated with these hormones, or translates these hormonal and molecular effects into performance. In addition, hen aging leads to a more rapid decline in productivity. This decline is often associated with a decrease in the concentration of oestrogen in hens (Taherkhani, Ghiasi, and Ebrahimi 2018). By stimulating the synthesis of egg yolk precursors, preventing a decrease in oestrogen levels may contribute to the sustainability of productivity in laying hens. Therefore, FEO might be a valuable mediator. Hence, we hypothesized that dietary supplementation with encapsulated FEO could enhance performance by modulating the reproductive hormonal response and the associated gene expressions during both the early and late laying phases of hens. This study aimed to test this hypothesis.

## 2 Materials and Methods

### 2.1 Animals, Housing, Experimental Design and Diets

This study was conducted at the Faculty of Agriculture, Department of Animal Science, Poultry Research and Application Facility. This study comprised two independent trials designed to evaluate the efficacy of encapsulated FEO during the early (29–40 weeks of age) and late (59–70 weeks of age) phases in laying hens. Commercial Atak‐S pullets (16–18 weeks of age) sourced from a local farm were reared at the University laying hen facility for 10 weeks before trial commencement. In each trial, 210 commercial Atak‐S laying hens with uniform body weight (BW) and similar performance were assigned to 3 treatments, each having 10 replicate cages containing 7 hens, over a 12‐week experimental period. Replicates were evenly distributed across the upper and lower cage levels to minimize the impact of cage placement. Dietary treatments in both early and late phase trials included a control (CON/FEO0) basal diet without FEO and basal diets containing FEO at 175 mg/kg (FEO175) and 350 mg/kg (FEO350), respectively. Previously, we reported that the FEO (≥99% purity, Birlikas, Izmir, Turkey) used in this study contained volatile components, including trans‐anethole (75.38%), limonene (9.52%), anisole (3.28%), carvone (3.13%) and *p*‐anisaldehyde (2.95%), along with linoleic acid (54.09%), oleic acid (33.44%), palmitic acid (6.29%), stearic acid (2.35%) and myristic acid (1.48%) (İpçak, Alçiçek, and Denli 2024). Encapsulation procedures were performed by revising the methods of Reineccius (1991) and Gouin (2004) to ensure a homogeneous mixture of FEO in the basal diets. This involved mixing FEO with a sodium alginate solution followed by dripping into a CaCl_2_ solution, ultimately encapsulating FEO within Na‐alginate gel beads. In both trials, the diets of the experimental groups were prepared at the feed production unit of the Department of Animal Science, Faculty of Agriculture, following the ATAK‐S management guidelines (TAEM 2015) and nutrient requirements of laying hens as reported by the National Research Council (NRC 1994). Before the trials, the proximate composition of the major ingredients in the diets was analysed. Table 1 presents the composition (g/kg), nutrient content (%), and metabolizable energy (kcal ME/kg) of the diets used in this study. The nutrient contents of the diets in both trials were determined using the Weende analysis method (AOAC 2000). The sugar and starch contents in the diets were determined using the Luff‐Schoorl technique and a two‐step process outlined in the TS 12232 and TS ISO 6493 standards, respectively (TSE 1997; 2004). Diets in mash form and water were provided ad libitum during the trials. All hens were housed in an environmentally controlled room with the temperature maintained at approximately 22°C ± 2°C and were kept in an enriched cage system. The enriched cage system comprised 3‐floors, with each floor containing 10 cages (80 cm length × 60 cm width × 77 cm height), and each cage was equipped with a perch, nest box and scratch pad. Controlled ventilation was maintained in the house throughout the trials, and a light cycle of 16 h of light and 8 h of darkness (16L:8D) was applied.

**TABLE 1:** Composition of ingredients (g/kg), nutrient content (%) and metabolizable energy (kcal/kg) of the diets formulated for laying hens.

Items	Early phase (29–40 weeks)			Late phase (59–70 weeks)		
	FEO0	FEO175	FEO350	FEO0	FEO175	FEO350
Corn	547.00	547.00	547.00	599.00	599.00	599.00
Soybean meal (46% CP)	180.00	180.00	180.00	223.00	223.0	223.0
Sunflower meal (32% CP)	120.00	120.00	120.00	31.00	31.00	31.00
Limestone (CaCO_3_)	85.00	85.00	85.00	94.00	94.00	94.00
Dicalciumphosphate	20.00	20.00	20.00	24.00	24.00	24.00
Sunflower oil (8800 kcal/kg)	40.00	40.00	40.00	20.00	20.00	20.00
dl‐Methionine	2.00	2.00	2.00	2.50	2.50	2.50
Vitamin–Mineral Premix^a^	2.50	2.50	2.50	2.50	2.50	2.50
NaCl	3.50	3.50	3.50	4.00	4.00	4.00
Encapsulated FEO	—	0.175	0.350	—	0.175	0.350
Analysed nutrients (%) and energy						
Dry matter	90.90	90.73	90.81	90.80	90.80	90.86
Crude protein	16.90	16.95	16.90	16.50	16.48	16.58
Ether extract	5.50	5.42	5.40	3.69	3.76	3.72
Crude fibre	4.79	4.80	4.75	2.93	2.90	2.98
Crude ash	13.65	13.80	13.70	14.71	14.50	14.52
Starch	37.24	37.43	37.32	40.43	40.21	40.12
Sugar	3.07	3.09	3.12	3.17	3.15	3.15
Metabolizable energy (ME poultry) (kcal/kg)	2658.46	2661.96	2655.75	2625.57	2621.17	2618.01
Calculated values						
Lysine (%)	0.80	0.80	0.80	0.85	0.85	0.85
Methionine + cystine	0.82	0.82	0.82	0.84	0.84	0.84
Ca (%)	3.71	3.71	3.71	4.14	4.14	4.14
Available P	0.45	0.45	0.45	0.52	0.52	0.52

^a^ Vitamin and mineral premix provided per 2.5 kg of diet: vitamin A, 12,000,000 IU; vitamin D3, 2500,000 IU; vitamin E, 30,000 mg; vitamin K3, 4000 mg; vitamin B1, 3000 mg; vitamin B2, 7000 mg; vitamin B6, 5000 mg; vitamin B12, 15 mg; CAL‐D pantothenate, 10,000 mg; biotin, 45 mg; folic acid, 1000 mg; canthaxanthin, 2500 mg; apo ester carotenoid, 500 mg; niacinamide, 3000 mg; choline chloride, 200,000 mg; Mn, 80,000 mg; Fe, 60,000 mg; Cu, 5000 mg; Zn, 60,000 mg; I, 1000 mg; Co, 200 mg; Se, 150 mg and CaCO_3_ 1390,000 mg.

### 2.2 Perfomance Measurements

All hens were individually weighed at the beginning and end of each trial to determine their initial and final BWs. Eggs were collected daily, and both the number and weight were recorded. Additionally, the number of cracked eggs was noted, and the cracked egg rate was calculated as a percentage of the total number of eggs produced. Hen‐day egg production (%) was determined weekly as the total number of eggs divided by 7 days. Average egg weight was calculated by dividing the total egg weight by the total number of eggs collected. The feed intake and FCR were recorded weekly. Egg mass was calculated by multiplying the average egg weight by egg production. The FCR was expressed as grams of feed intake per gram of egg mass. Performance parameters for each trial were reported on a hen‐day basis over 12 weeks, divided into 3 consecutive 4‐week periods (29–32 weeks, 33–36 weeks and 37–40 weeks for the early laying phase; 59–62 weeks, 63–66 weeks and 67–70 weeks for the late laying phase), as well as for the total study intervals. Mortality was recorded daily in both trials.

### 2.3 Sampling Procedures for Subsequent Analyses

At the end of both trials (at 40 and 70 weeks of layers age, respectively), 10 birds from each experimental group (1 hen per replicate) were randomly selected. Blood samples were obtained from the jugular vein using sterilized needles and syringes and then collected into tubes. The blood samples were centrifuged at 4000 rpm for 10 min at room temperature, and the resulting serum was collected and stored at −20°C until further analysis. Subsequently, the hens were anaesthetized with an intraperitoneal injection of ketamine (23 mg/kg BW) and humanely euthanized via rapid decapitation. The reproductive organs were carefully excised and immediately weighed. Ovary samples were dissected, snap‐frozen in liquid nitrogen and stored at −80°C until total RNA isolation.

### 2.4 Assessing Follicular Development

Follicular grading was performed according to the method described by Johnson and Woods (2009). The number and weight of pre‐hierarchical follicles, including small white follicles (SWFs, 1–3.9 mm), large white follicles (LWFs, 4–4.9 mm) and small yellow follicles (SYFs, 5–8 mm), as well as the number and weight of hierarchical follicles (F1–F5/F6, >9 mm), were measured. These were reported as the total number and weight of both the pre‐hierarchical and hierarchical follicles. The classification of pre‐hierarchical and hierarchical follicles was based on their weight, diameter and order of ovulation. If the weight difference between the two hierarchical follicles was less than 1 g, they were considered to be of the same rank in the hierarchy (Hocking 1996).

### 2.5 Determination of Serum Hormone Profile

In both trials, serum levels of oestradiol (*E*2), FSH, LH, prolactin, progesterone and testosterone were analysed using the electrochemiluminescence (ECLIA) assay method with commercial assay kits (Elecsys, Roche Diagnostics, Germany) and an automatic biochemical analyser (Cobas e 601, Roche Diagnostics, Germany), following the manufacturer's guidelines. Moreover, intra‐ and inter‐assay coefficients of variation (CV) were calculated to assess the precision of hormone measurements across different dietary treatments and laying phases. CV values <15% were considered acceptable (Food and Drug Administration, 2018).

### 2.6 RNA Isolation and cDNA Synthesis

In both trials, total RNA from ovarian and blood samples was isolated using the High Pure RNA Isolation Kit (Roche Diagnostics, Switzerland), according to the manufacturer's protocol. After isolation, RNA quality and quantity (OD260/280) were determined using a NanoDrop 2000 spectrophotometer (Thermo Scientific, Wilmington, DE, USA). Only RNA samples with an OD260/OD280 ratio between 1.8 and 2.1 were used for cDNA analysis. Besides the concentration determination, the quality of isolated RNA samples was assessed by agarose gel electrophoresis. The presence of two distinct ribosomal RNA (28S and 18S rRNA) bands on the gel indicated that the RNA samples were of good quality and integrity. For cDNA preparation, 1000 ng of total RNA from each sample was reverse‐transcribed to cDNA using the Thermo RevertAid RT Reverse Transcription Kit (Thermo Scientific, Wilmington, DE, USA), on the basis of the provided directions. Prior to polymerase chain reaction (PCR) reaction, RNAs were mixed with random hexamer (0.5 µL), oligo dT (0.5 µL) and nuclease free H_2_O (12 µL), incubated at 65°C for 5 min and then cooled on ice. Subsequently, the reaction mixture consisted of 4 µL 5× reaction buffer, 1 µL RevertAid (200 U/µL) reverse transcriptase, 1 µL RiboLock RNase Inhibitor (20 U/µL), 2 µL dNTP Mix (10 mM) and 12 µL RNA sample, with a total volume of 20 µL for a single sample. The Thermocycler (Bio‐Rad Laboratories, USA) protocol was conducted as follows: priming at 25°C for 5 min, reverse transcription at 42°C for 60 min, RT inactivation at 70°C for 5 min, and then holding at 4°C. The cDNAs were then stored at −20°C.

### 2.7 Quantitative Real‐Time PCR (qRT‐PCR) Analysis

The following genes related to the reproductive axis in *Gallus gallus* were examined in the blood and ovarian tissue to determine target gene expression: FSH receptor (FSHR), prolactin receptor (PRLR), steroidogenic acute regulatory protein (STAR) and oestrogen receptor 2 (ESR2). The β‐actin (ACTB) housekeeping gene was used as the internal control for normalization. Primers were designed using GenBank from the National Centre for Biotechnology Information (NCBI) and ENSEMBL (Table 2), and their specificity was verified using the PRIMER BLAST programme to ensure the generation of a unique amplicon. qRT‐PCR analysis was performed using a Roche LightCycler 480 II system (Roche Diagnostics, Basel, Switzerland). Each reaction, with a total volume of 20 µL, included 3 µL of cDNA, 10 µL of LightCycler 480 SYBR Green I Master Mix, 5.8 µL of PCR‐grade water, 0.6 µL of the forward primer and 0.6 µL of the reverse primer, both at a concentration of 10 µmol/L. The amplification conditions were set as follows: (1) initial denaturation at 95°C for 5 min; (2) 45 cycles of denaturation at 95°C for 10 s, annealing at 56°C for 15 s and elongation at 72°C for 10 s; (3) melting curve analysis involving a gradual increase in temperature from 60°C to 97°C in increments of 2.5°C every 2.5 s; and (4) final cooling at 40°C for 30 s. Data analysis utilized both Absolute Quantification and Advanced Relative Quantification methods, with the fold‐change method applied to interpret the results, following Lee et al. (2006). Using this approach, the cycle threshold (Ct) values of the target genes were normalized to those of the housekeeping gene. Fold‐change values were calculated by comparing normalized values with those of the control group.

**TABLE 2:** Forward (F) and reverse (R) primers of genes.

Genes	Accession number	Primer sequence (5′–3′)	Primer position	Product length (bp)
FSHR	NM_205079.2	F: TGAGGCAAACTTCACCTATCC R: TGCTGGAGACATGCTACATATT	918–938 1014–993	97
PRLR	XM_046934786.1	F: CTCCACCTCTATTAGCTGATGC R: CAGATATTGTCTCCCACTCTTCC	229–250 324–302	96
STAR	NM_204686.3	F: CGAGCAGCAGGGATTTATCA R: ATGCTAAGAAGCCACGTCAA	684–703 788–769	105
ESR2	NM_001396358.1	F: TGCTCTGGTGTGGGTTATTG R: CAGATGCTCCATGCCCTTATTA	1635–1654 1758–1737	124
ACTB	NM_205518.1	F:TGGGCCAGAAAGACAGCTAC R: CCGTGTTCAATGGGGTACTT	208–227 289–270	82

Abbreviations: ACTB, β‐actin; ESR2, oestrogen receptor 2; FSHR, follicle‐stimulating hormone receptor; PRLR: prolactin receptor; STAR, steroidogenic acute regulatory protein.

### 2.8 Statistical Analysis

The experimental data were initially assessed for normality, and differences between group means were analysed using the general linear model (GLM)–ANOVA procedure, implemented in SPSS statistical software (Version 22.0, SPSS Inc., 2013) (SPSS 2013). Duncan's multiple range test was applied to assess the significance of differences between groups, with a level of *p* < 0.05, considered significant and 0.05 < *p* < 0.10 considered indicative of a trend. Orthogonal contrasts were used to investigate linear and quadratic response patterns related to dietary FEO inclusion levels. The chi‐square test was applied to determine differences in mortality rates between the groups. Finally, Pearson's correlation analysis was performed to evaluate the association between the gene expression levels and egg production in the FEO‐supplemented groups.

## 3 Results

### 3.1 Productive and Laying Performance

The effects of dietary FEO levels on productive and laying performance during the early laying phase are presented in Table 3. The data showed that growth was not affected when hens were fed a diet supplemented with FEO compared with the control during the early laying phase (*p* > 0.05). Hen‐day egg production significantly and linearly increased with the FEO350 treatment compared to the control group and FEO175 treatments throughout weeks 29–32, 33–36 and over the total period (*p* < 0.05). Egg production (81.84% vs. 85.63% for control vs. FEO350) in hens treated with 350 mg/kg FEO increased by 4.63% compared to that in the control group (*p* < 0.05). Moreover, increasing levels of FEO significantly enhanced egg weight and egg mass during the age period of 33–36 weeks (linear effect, *p* < 0.05). All groups supplemented with FEO had significantly higher egg weights than the control group (*p* < 0.05). Specifically, the egg mass in the FEO350 treatment group was significantly higher than that in the control group and exceeded that in the FEO175 group (*p *< 0.05). Additionally, during the age period of 37–40 weeks, as well as over the total period, feed intake decreased in both linear (*p_lin_* < 0.05) and quadratic (*p_quad_* < 0.05) manners with increasing FEO inclusion levels, with the FEO350 group exhibiting lower feed intake than the control. The dietary inclusion of 350 mg/kg FEO also improved the FCR (2.13 vs. 2.27 for FEO350 vs. control) and demonstrated quadratic patterns of decrease with increasing FEO inclusion level over the total period (*p* < 0.05). Furthermore, there was no significant difference in the mortality rates between the experimental treatments during the early phase (*p* > 0.05).

**TABLE 3:** Effect of dietary fennel seed essential oil (FEO) inclusion levels on productive and laying performance during the early laying phase.

	FEO^†^ (mg/kg)				*p* values		
Item	0	175	350	Plood SEM^‡^	Main effect	Linear effect	Quadratic effect
Initial BW (g)	1442.14	1403.22	1423.65	7.902	0.129	0.315	0.076
Final BW (g)	1735.60	1745.60	1742.20	36.868	0.995	0.947	0.938
Hen‐day egg production (%)							
29–32 weeks	76.35^b^	80.27^ab^	84.29^a^	1.167	0.018	0.005	0.985
33–36 weeks	84.33^b^	83.41^b^	90.40^a^	1.240	0.041	0.042	0.124
37–40 weeks	85.71	80.11	82.22	1.400	0.276	0.319	0.192
Total (29–40 weeks)	81.84^b^	81.26^b^	85.63^a^	0.750	0.035	0.044	0.108
Egg weight (g)							
29–32 weeks	59.14	59.68	59.84	0.179	0.252	0.086	0.644
33–36 weeks	60.00	60.62	60.98	0.176	0.056	0.008	0.770
37–40 weeks	60.05	60.75	60.82	0.214	0.295	0.105	0.494
Total (29–40 weeks)	59.65^b^	60.28^a^	60.55^a^	0.116	0.005	0.001	0.509
Egg mass (g/hen/d)							
29–32 weeks	45.23^b^	47.94^ab^	50.14^a^	0.768	0.030	0.009	0.869
33–36 weeks	50.60	50.63	54.75	0.837	0.074	0.047	0.253
37–40 weeks	51.31	48.77	49.92	0.914	0.546	0.543	0.347
Total (29–40 weeks)	48.72^b^	49.13^b^	51.60^a^	0.495	0.040	0.019	0.330
Feed intake (g/hen/d)							
29–32 weeks	117.30	121.81	121.27	1.060	0.132	0.993	0.045
33–36 weeks	118.31	119.24	118.59	1.173	0.079	0.102	0.125
37–40 weeks	121.39^b^	117.03^b^	112.74^a^	1.780	<0.001	<0.001	0.003
Total (29–40 weeks)	119.52^b^	119.92^b^	116.89^a^	0.738	<0.001	<0.001	<0.001
Feed conversion ratio							
29–32 weeks	2.57	2.56	2.42	0.041	0.359	0.192	0.579
33–36 weeks	2.34	2.36	2.18	0.044	0.332	0.242	0.360
37–40 weeks	2.37	2.40	2.25	0.049	0.699	0.407	0.899
Total (29–40 weeks)	2.43^b^	2.44^b^	2.26^a^	0.027	0.031	0.191	<0.001
Mortality (%)	1.40	1.40	0.00	—	0.364	—	—

*Note*: Mean values with different superscripts (a, b) within the same row are significantly different (*p* < 0.05).

Abbreviation: BW, body weight.

^†^ The control group was fed basal diets without the addition of encapsulated FEO (FEO0), and the treatment groups were fed basal diets supplemented with 175 and 350 mg encapsulated FEO/kg (FEO175 and FEO350, respectively).

^‡^ Pooled standard error of the mean.

The effects of dietary FEO levels on productive and laying performance during the late laying phase are shown in Table 4. FEO supplementation did not affect the final BW of laying hens during the late laying phase (*p* > 0.05). Hen‐day egg production (75.82% vs. 80.33% for control vs. FEO350) linearly increased with increasing FEO levels, with FEO350 treatment improving egg production by 5.95% compared to the control over the total period (*p* < 0.05). Egg weight exhibited a quadratic decrease in groups supplemented with 175 mg/kg FEO (*p* < 0.05), whereas the egg weight values were similar between the FEO350 and control groups. The FEO350 treated group demonstrated both linear (*p_lin_* < 0.05) and quadratic (*p_quad_* < 0.05) increases in egg mass from the 59 to 62 weeks of age and over the total period (*p* < 0.05). Additionally, egg mass linearly increased with FEO350 treatment from 63 to 66 weeks of age (*p* < 0.05). Supplemental FEO had no effect on feed intake during the total period (*p* > 0.05). However, the dietary inclusion of FEO significantly and linearly decreased the FCR (2.29 vs. 2.42 for FEO350 vs. control) during the late phase (*p* < 0.05). No differences in mortality were observed between the treatment groups during the late laying phase (*p* > 0.05).

**TABLE 4:** Effect of dietary fennel seed essential oil (FEO) inclusion levels on productive and laying performance during the late laying phase.

	FEO^†^ (mg/kg)				*p* values		
Item	0	175	350	Plood SEM^‡^	Main effect	Linear effect	Quadratic effect
Initial BW (g)	1745.60	1746.42	1780.53	24.496	0.809	0.567	0.754
Final BW (g)	1762.80	1733.60	1775.20	40.263	0.922	0.909	0.706
Hen‐day egg production (%)							
59–62 weeks	77.24	76.59	79.09	0.904	0.625	0.343	0.826
63–66 weeks	78.57^b^	81.47^ab^	83.72^a^	0.805	0.033	0.015	0.840
67–70 weeks	71.44^b^	74.24^ab^	77.10^a^	1.029	0.029	0.013	0.796
Total (59–70 weeks)	75.82^b^	77.50^ab^	80.33^a^	0.549	0.046	0.016	0.997
Egg weight (g)							
59–62 weeks	61.98^a^	61.13^b^	63.10^a^	0.167	0.019	0.571	0.006
63–66 weeks	62.49^a^	61.49^b^	62.36^a^	0.132	0.003	0.721	<0.001
67–70 week	62.97	62.18	62.45	0.178	0.173	0.561	0.102
Total (59–70 weeks)	62.48^a^	61.69^b^	62.42^a^	0.092	<0.001	0.848	<0.001
Egg mass (g/hen/d)							
59–62 weeks	47.97^ab^	46.52^b^	50.44^a^	0.600	0.010	0.070	0.012
63–66 weeks	49.20^b^	49.48^b^	52.33^a^	0.511	0.039	0.022	0.259
67–70 weeks	45.62	46.89	47.60	0.652	0.563	0.661	0.329
Total (59–70 weeks)	47.40^b^	48.18^b^	50.13^a^	0.348	0.002	0.016	0.009
Feed intake (g/hen/d)							
59–62 weeks	122.06	115.77	119.20	1.447	0.186	0.174	0.206
63–66 weeks	113.90	113.14	113.41	1.207	0.967	0.870	0.844
67–70 weeks	112.46	113.38	111.18	1.192	0.588	0.654	0.354
Total (59–70 weeks)	116.14	114.12	115.02	0.938	0.496	0.395	0.408
Feed conversion ratio							
59–62 weeks	2.54	2.49	2.37	0.033	0.156	0.065	0.630
63–66 weeks	2.32	2.28	2.17	0.039	0.157	0.092	0.292
67–70 weeks	2.47	2.42	2.39	0.035	0.518	0.480	0.366
Total (59–70 weeks)	2.45^b^	2.37^ab^	2.29^a^	0.021	0.044	0.009	0.768
Mortality (%)	2.90	1.40	1.40	—	0.601	—	—

*Note*: Mean values with different superscripts (a, b) within the same row are significantly different (*p* < 0.05).

Abbreviation: BW, body weight.

^†^ The control group was fed basal diets without the addition of encapsulated FEO (FEO0), and the treatment groups were fed basal diets supplemented with 175 and 350 mg encapsulated FEO/kg (FEO175 and FEO350, respectively).

^‡^ Pooled standard error of the mean.

### 3.2 Cracked Egg Rate and Follicle Development

**FIGURE 1:**
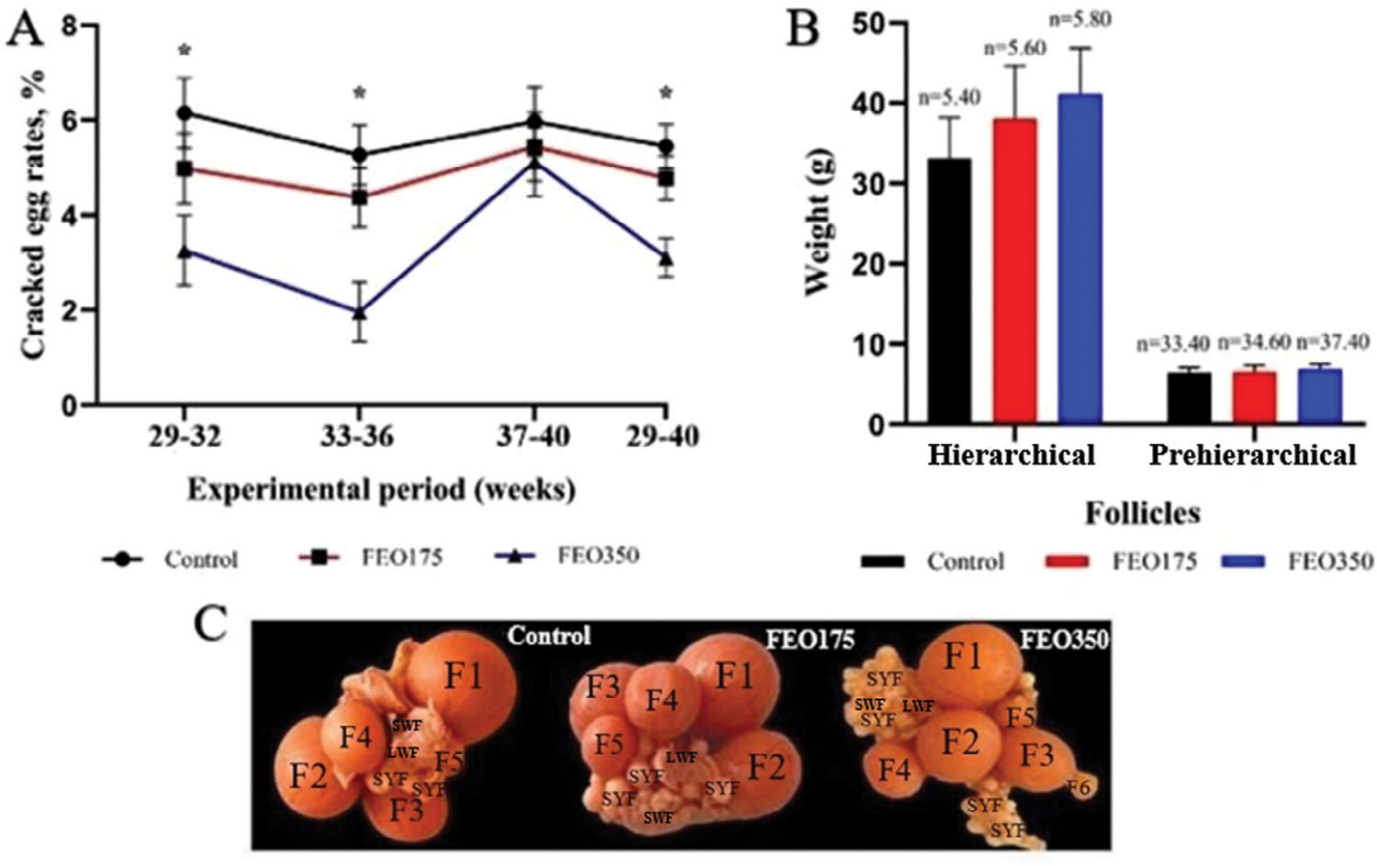
Effect of dietary FEO inclusion levels on cracked egg rates (A) as well as the number and weight of follicles (B) and follicular development (C) during the early laying phase. The control group was fed basal diets without the addition of encapsulated FEO (FEO0), whereas the treatment groups were fed basal diets supplemented with 175 and 350 mg of encapsulated FEO/kg (FEO175 and FEO350, respectively). Pre‐hierarchical follicles include small white follicles (SWFs), large white follicles (LWFs) and small yellow follicles (SYFs). Hierarchical follicles are referred to as F1 to F5/F6. F1 represents the biggest follicle, F2 the next largest and so forth. *n* indicates the number of each type of follicles could be identified from all the 10 ovaries in each group. Data are presented as LSM (least squares means), with error bars representing ± SEM (standard error of the mean). Asterisks indicate statistically significant differences between the groups (*p* < 0.05).

Increasing levels of FEO resulted in a decrease in the rate of cracked eggs, with hens fed 350 mg/kg FEO showing lower rates of cracked eggs compared to both the control and FEO175 groups during the 29–32, 33–36 weeks of age and over the total period (*p* < 0.05; Figure 1). However, the number and weight of hierarchical and pre‐hierarchical follicles were not significantly influenced by supplemental FEO, despite numerical differences (*p* > 0.05).

**FIGURE 2:**
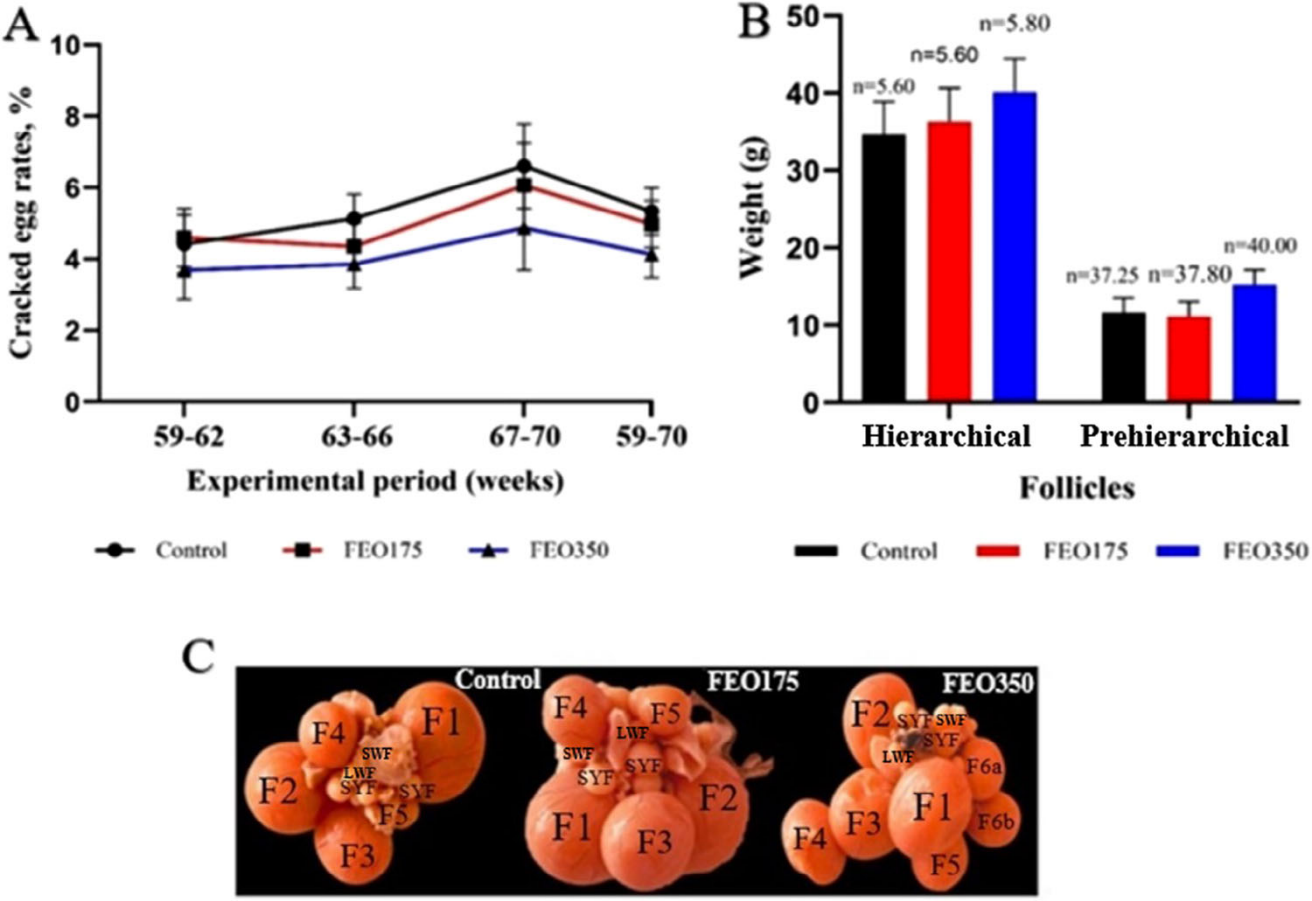
Effect of dietary FEO inclusion levels on cracked egg rates (A) as well as the number and weight of hierarchical follicles (B) and follicular development (C) during the late laying phase. The control group was fed basal diets without the addition of encapsulated FEO (FEO0), whereas the treatment groups were fed basal diets supplemented with 175 and 350 mg of encapsulated FEO/kg (FEO175 and FEO350, respectively). Pre‐hierarchical follicles include small white follicles (SWFs), large white follicles (LWFs) and small yellow follicles (SYFs). Hierarchical follicles are referred to as F1 to F5/F6. F1 represents the biggest follicle, F2 the next largest and so forth. The notation ‘a‐b’ represents follicles of similar size, possibly indicating double‐yolk potential. *n* indicates the number of each type of follicles could be identified from all the 10 ovaries in each group. Data are presented as LSM, with error bars representing ± SEM.

In the FEO‐supplemented groups, particularly in the FEO350 treatment group, there was a numerical decrease in the rate of cracked eggs and visible improvements in follicles, along with a numerical increase in follicle numbers and weights. However, these differences between the treatment groups were not statistically significant (*p* > 0.05; Figure 2).

### 3.3 Serum Reproductive Hormone Profile

The serum reproductive hormone profiles of the hens during the early and late laying phases are provided in Table 5. In the early phase of hens, *E*2 was significantly and linearly increased by the FEO350 treatment compared to the control and FEO175 treatments (*p* < 0.05). All groups supplemented with FEO had higher FSH concentrations than the control (*p* < 0.05). LH concentrations increased both linearly and quadratically with increasing FEO inclusion levels, with FEO350 showing higher values than the other groups (*p* < 0.05). There was no significant difference in prolactin levels between the experimental treatments (*p* > 0.05). Progesterone levels increased in all FEO‐treated hens, and the testosterone level in the FEO175 treatment was higher than that in the control and FEO350 groups (*p* < 0.05). In the late phase of hens, treatment with FEO175 and FEO350 linearly increased *E*2 and FSH concentrations compared with the control (*p* < 0.05). LH concentration increased linearly pattern with increasing FEO level, with the FEO350 treatment displaying the highest LH value (*p* < 0.05). The levels of prolactin were not influenced by dietary FEO inclusion levels (*p* > 0.05). An increase in FEO levels led to an increase in progesterone concentration in both a linear (*p_lin_* < 0.05) and quadratic (*p_quad_* < 0.05) manner. The FEO175 treatment resulted in the highest testosterone level compared to the other treatment groups (*p* < 0.05). Additionally, the calculated intra‐assay CVs ranged from 1.43% to 10.00%, whereas the inter‐assay CVs ranged from 3.03% to 15.00%, indicating a consistent and reliable assay performance across both experimental phases.

**TABLE 5:** Effect of dietary FEO inclusion levels on serum reproductive hormone profiles during the early and late laying phase.

	FEO^†^ (mg/kg)				*p* values			Intra‐assay CV (%)			Inter‐assay CV (%)		
								FEO^†^ (mg/kg)			FEO^†^ (mg/kg)		
Item	0	175	350	Plood SEM^‡^	Main effect	Linear effect	Quadratic effect	0	175	350	0	175	350
Early laying phase													
*E*2 (pg/mL)	200.00^b^	240.08^ab^	260.08^a^	9.914	0.024	0.023	0.584	8.74	5.97	5.85	13.95	9.81	10.61
FSH (mIU/mL)	0.29^b^	0.31^a^	0.32^a^	0.005	0.003	0.001	0.323	5.86	4.19	1.56	5.17	5.48	3.03
LH (mIU/mL)	0.09^c^	0.11^b^	0.15^a^	0.007	<0.001	<0.001	0.043	6.67	7.27	6.00	10.00	15.00	12.50
Prolactin (ng/mL)	0.05	0.05	0.06	0.002	0.701	0.416	0.889	10.00	6.00	8.00	14.00	6.82	12.50
Progesterone (ng/mL)	1.38^b^	1.59^b^	2.52^a^	0.148	<0.001	0.001	0.017	9.88	7.61	7.99	13.70	12.64	12.60
Testesterone (ng/dL)	0.04^c^	0.09^a^	0.07^b^	0.006	<0.001	<0.001	<0.001	7.50	7.78	7.14	12.12	12.50	14.89
Late laying phase													
*E*2 (pg/mL)	195.70^b^	249.10^a^	267.20^a^	11.943	0.024	0.009	0.397	7.49	3.85	6.85	13.95	9.81	10.61
FSH (mIU/mL)	0.30^b^	0.32^a^	0.33^a^	0.005	0.011	0.004	0.503	3.67	6.25	5.63	5.17	5.48	3.03
LH (mIU/mL)	0.11^c^	0.14^b^	0.16^a^	0.007	<0.001	<0.001	0.577	3.63	9.29	7.86	10.00	15.00	12.50
Prolactin (ng/mL)	0.05	0.04	0.08	0.002	0.225	0.105	0.585	6.00	2.50	7.50	14.00	6.82	12.50
Progesterone (ng/mL)	0.30^c^	0.37^b^	1.86^a^	0.192	<0.001	<0.001	<0.001	4.30	1.89	1.43	13.70	12.64	12.60
Testesterone (ng/dL)	0.03^b^	0.07^a^	0.03^b^	0.006	<0.001	<0.001	<0.001	6.67	8.57	10.00	12.12	12.50	14.89

*Note*: Mean values with different superscripts (a, b) within the same row are significantly different (*p* < 0.05).

Abbreviations: CV, coefficient of variation; *E*2, oestradiol; FSH, follicle‐stimulating hormone; LH, Luteinizing hormone.

^†^ The control group was fed basal diets without the addition of encapsulated FEO (FEO0), and the treatment groups were fed basal diets supplemented with 175 and 350 mg encapsulated FEO/kg (FEO175 and FEO350, respectively).

^‡^ Pooled standard error of the mean.

### 3.4 Gene Expression Profiles Associated With Reproductive Axis

**FIGURE 3:**
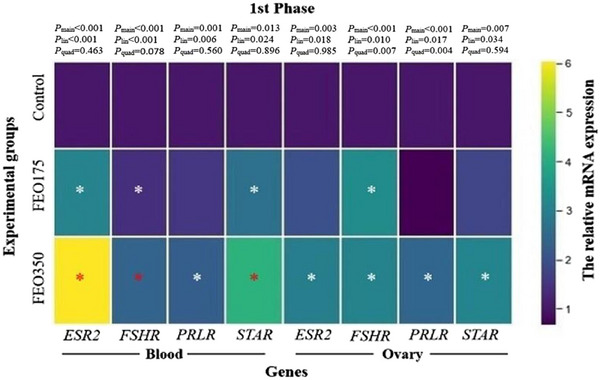
A heatmap illustrating the effect of dietary FEO inclusion levels on blood and ovarian reproductive axis‐related gene expression during the early laying phase. The control group was fed basal diets without the addition of encapsulated FEO (FEO0), whereas the treatment groups were fed basal diets supplemented with 175 and 350 mg of encapsulated FEO/kg (FEO175 and FEO350, respectively). The colour intensity indicates up‐ or down‐regulated gene mRNA expression levels in each matrix. The asterisk represents significant differences between the control and FEO‐supplemented groups, with the colour of the asterisk indicating a significant difference between the FEO‐supplemented groups. ESR2, oestrogen receptor 2 (or ERβ); FSHR, follicle stimulating hormone receptor; PRLR: prolactin receptor; STAR, steroidogenic acute regulatory protein. *p* < 0.05.

The relative expression levels of genes related to reproductive axis in blood and ovaries during the early laying phase are shown in Figure 3. Increasing levels of FEO enhanced the expression levels of ESR2 (3.10‐ and 6.04‐fold, for FEO175 and FEO350, respectively) and FSHR (1.43‐ and 2.38‐fold, for FEO175 and FEO350, respectively) in the blood compared to the control (*p* < 0.05). Treatment with FEO350 increased the expression of PRLR and STAR in the both blood and ovaries with the other groups (*p* < 0.05). Ovarian FSHR expression was higher in the FEO175 and FEO350 treatments than that in the control (3.26‐ and 3.07‐fold, respectively; *p* < 0.05). Additionally, the highest expression of ovarian ESR2 was observed in FEO350 treatment groups (*p* < 0.05).

**FIGURE 4:**
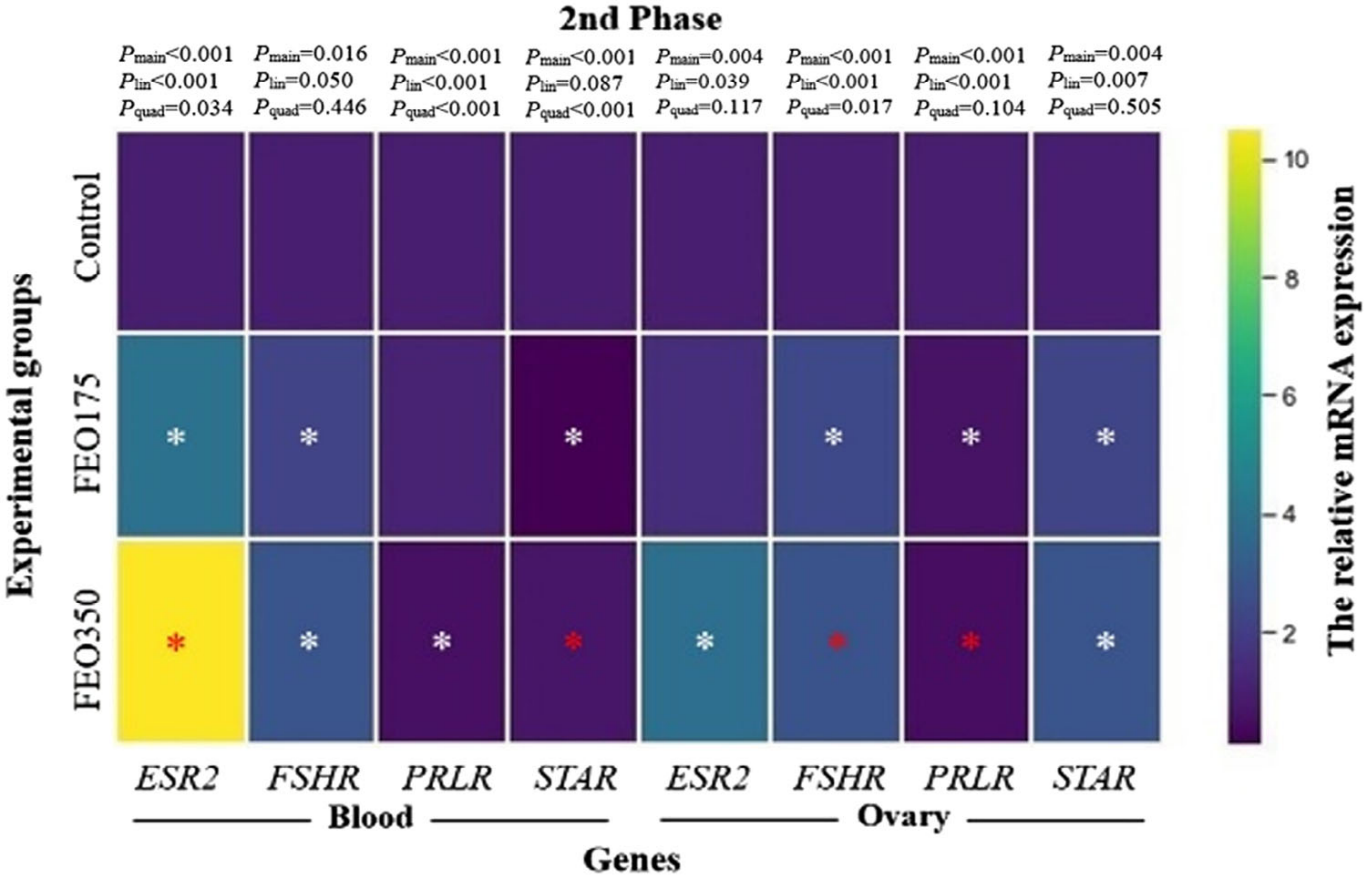
A heatmap illustrating the effect of dietary FEO inclusion levels on blood and ovarian reproductive axis‐related gene expression during the late laying phase. Similar to Figure 3, the control group was fed basal diets without the addition of encapsulated FEO (FEO0), whereas the treatment groups were fed basal diets supplemented with 175 and 350 mg of encapsulated FEO/kg (FEO175 and FEO350, respectively). The colour intensity represents up‐ or down‐regulated gene mRNA expression levels in each matrix. The asterisk indicates significant differences between the control and FEO‐supplemented groups, with the colour of the asterisk indicating a significant difference between the FEO‐supplemented groups. ESR2, oestrogen receptor 2 (or ERβ); FSHR, follicle stimulating hormone receptor; PRLR, prolactin receptor; STAR, steroidogenic acute regulatory protein. *p* < 0.05.

The relative expression levels of the genes associated with the reproductive axis in the blood and ovaries during the late laying phase are shown in Figure 4. Treatment with FEO175 and FEO350 elevated ESR2 expression in both the blood (4.04‐ and 10.50‐fold, respectively) and ovaries (1.48‐ and 3.82‐fold, respectively) compared to the control (*p* < 0.05). FSHR expression in the blood and STAR expression in the ovaries were higher in the FEO‐supplemented groups than in the control (*p* < 0.05). The lowest expression of PRLR in both the blood and ovaries was observed with treatment with FEO350 (0.54‐ and 0.44‐fold, respectively; *p* < 0.05). Furthermore, treatment with FEO175 and FEO350 reduced STAR expression in the blood (0.11‐ and 0.69‐fold, respectively) but increased ovarian FSHR expression (2.40‐ and 2.78‐fold, respectively) with increasing FEO inclusion levels (*p* < 0.05).

### 3.5 Association Between Egg Production and Gene Expression

**FIGURE 5:**
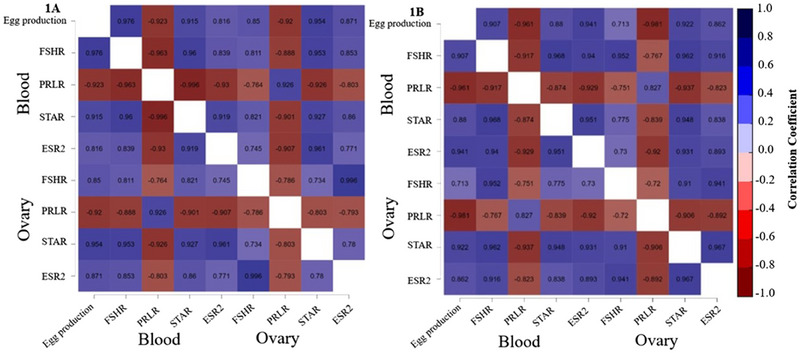
Association between blood and ovarian reproductive axis‐related genes mRNA expression levels and egg production in hens during the early laying phase. (A) Correlation between egg production and mRNA expression levels of reproductive axis‐related genes in the FEO175 treatment group. (B) Correlation between egg production and mRNA expression levels of reproductive axis‐related genes in the FEO350 treatment group. ESR2, oestrogen receptor 2 (or ERβ); FSHR, follicle‐stimulating hormone receptor; PRLR, prolactin receptor; *r*, Pearson correlation coefficient value; STAR, steroidogenic acute regulatory protein. *p* < 0.05.

The correlations between FSHR, PRLR, STAR and ESR2 levels in the blood and ovaries and egg production during the early laying phase are presented in Figure 5. In the FEO175 group (1A), correlation analysis revealed significant positive associations between egg production and blood levels of FSHR (*r* = 0.98, *p* < 0.05), STAR (*r* = 0.92, *p* < 0.05) and ESR2 (*r* = 0.82, *p* < 0.05) and a significant negative correlation with the blood PRLR expression level (*r* = −0.92, *p* < 0.05). Additionally, a significant positive correlation was observed between egg production and ovarian STAR expression level (*r* = 0.95, *p *< 0.05), and a significant negative correlation with ovarian PRLR expression levels (*r* = −0.92, *p *< 0.05). However, there were no significant correlations between egg production and the ovarian expression levels of FSHR (*r* = 0.85, *p* > 0.05) and ESR2 (*r* = 0.87, *p* > 0.05) in the FEO175 group. In the FEO350 group (1B), there were significant positive correlations between egg production and blood levels of FSHR (*r* = 0.91, *p* < 0.05), STAR (*r* = 0.88, *p* < 0.05) and ESR2 (*r* = 0.94, *p* < 0.05), and a significant negative correlation with PRLR expression levels (*r* = −0.96, *p* < 0.05). Similarly, significant positive correlations were found between egg production and ovarian STAR expression levels (*r* = 0.92, *p* < 0.05), and a significant negative correlation with PRLR expression levels (*r* = −0.98, *p* < 0.05). No significant relationships were identified between egg production and ovarian FSHR (*r* = 0.71, *p* > 0.05) or ESR2 (*r* = 0.86, *p* > 0.05) expression levels in the FEO350 group.

**FIGURE 6:**
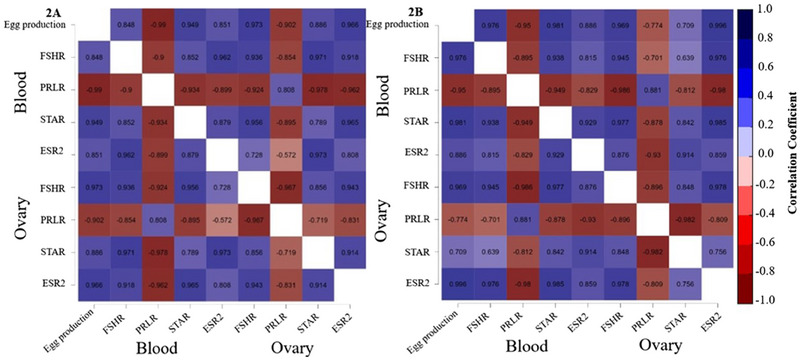
Association between blood and ovarian reproductive axis‐related genes mRNA expression levels and egg production in hens during the late laying phase. (A) Correlation between egg production and mRNA expression levels of reproductive axis‐related genes in the FEO175 treatment group. (B) Correlation between egg production and mRNA expression levels of reproductive axis‐related genes in the FEO350 treatment group. ESR2, oestrogen receptor 2 (or ERβ); FSHR, follicle‐stimulating hormone receptor; PRLR, prolactin receptor; *r*, Pearson correlation coefficient value; STAR, steroidogenic acute regulatory protein. *p* < 0.05.

The correlations between FSHR, PRLR, STAR and ESR2 levels in the blood and ovaries and egg production during the late laying phase are shown in Figure 6. In the FEO175 group (2A), significant positive correlations were observed between egg production and the expression levels of blood FSHR (*r* = 0.85, *p* < 0.05), STAR (*r* = 0.95, *p* < 0.05) and ESR2 (*r* = 0.85, *p* < 0.05), and significant negative correlations were observed with PRLR expression levels (*r* = −0.99, *p* < 0.05). Additionally, significant positive correlations were found between egg production and the expression levels of ovarian FSHR (*r* = 0.97, *p* < 0.05), STAR (*r* = 0.89, *p* < 0.05) and ESR2 (*r* = 0.97, *p* < 0.05), and significant negative correlations with the PRLR expression level (*r* = −0.90, *p* < 0.05). Furthermore, in the FEO350 group (2B), significant direct positive correlations were observed between egg production and the expression levels of blood FSHR (*r* = 0.98, *p* < 0.05), STAR (*r* = 0.98, *p* < 0.05) and ESR2 (*r* = 0.89, *p* < 0.05), and a significant negative correlation with the PRLR expression level (*r* = −0.95, *p* < 0.05). Additionally, a significant positive correlation was found between egg production and expression levels of ovarian FSHR (*r* = 0.97, *p *< 0.05) and ESR2 (*r* = 0.99, *p* < 0.05). However, no significant correlation was observed between egg production and the expression levels of PRLR (*r* = −0.77, *p* > 0.05) and STAR (*r* = 0.71, *p *> 0.05).

## 4 Discussion

Egg production is a key indicator of laying performance in hens (Ma et al. 2024). In the current study, we found that increasing FEO levels linearly enhanced egg production during the early laying phase. This result is consistent with those reported by Samantaray and Nayak (2022). Additionally, Abou‐Elkhair, Selim, and Hussein (2018) observed that feeding fennel seed powder at 5 g/kg increased egg production in hens aged 32–40 weeks. Contrary to our findings, Beshara (2018) found that anise seeds as a source of anethole did not affect egg production. However, owing to the limited number of reports on the effect of FEO on laying hens, comparisons were made with other studies employing similar dietary phytogenics such as aromatic plants, herbs, spices, essential oils and their bioactive components. The beneficial effects of the dietary inclusion of peppermint oil (Abdel‐Wareth and Lohakare 2020), lavender essential oil (Torki, Mohebbifar, and Mohammadi 2021) and Shudi Erzi San (Zhang et al. 2022) on egg production have been previously reported in the early laying phase of hens. However, it has been suggested that mint essential oil (250 mg/kg) or a combination of lavender and mint essential oils (250 mg/kg each) decreased egg production (Torki, Mohebbifar, and Mohammadi 2021). In contrast, Brouklogiannis et al. (2023) reported that in laying hens, productive performance is closely linked to ovarian function, and ovarian aging can affect performance and lifespan. Taherkhani, Ghiasi, and Ebrahimi (2018) stated that decreased egg production is associated with aging, and that this decline is typically correlated with a decrease in the concentration of oestrogen. Our findings demonstrated that FEO supplementation also increased egg production during the late laying phase. The findings of the present study regarding the percentage of egg production in the FEO‐supplemented groups are in agreement with those reported by Taherkhani, Ghiasi, and Ebrahimi (2018). However, in a recent study, Xiao et al. (2022) observed that essential oils (carvacrol, thymol, cinnamaldehyde and carrier nanoscale silica) did not affect egg production. This result was also consistent with data obtained from dried peppermint leaves (Abdel‐Wareth and Lohakare 2014) and Enviva essential oil (Ding et al. 2017).

In both trials, increased egg production may have resulted from the active components of FEO. FEO contains a significant amount (75.38%) of anethole (İpçak, Alçiçek, and Denli 2024), which is the main active component and serves as a polymer with phytoestrogenic properties (Badgujar, Patel, and Bandivdekar 2014). Owing to their structural similarity to oestradiol (*E*2), which is the most biologically active form of oestrogen, phytoestrogens can act as oestrogen (Naz 2004). Therefore, anethole interacts with ER, potentially contributing to the maintenance of the ovulatory cycle and functionality of the reproductive system in laying hens, thereby positively affecting egg production (Beshara 2018). Another contributing factor could be the strong antioxidant activity of FEO (Shahat et al. 2011), which might alleviate the potential negative effects of the chickenhouse environment. Thus, FEO might have played a role in the observed improvement in egg production. In addition, despite the increasing age of the layers, FEO supplementation continued to improve egg production compared with the control. This result could be attributed to the preservation of oestrogen levels, which are important for maintaining the ovulatory cycle in the body of the layers. Anethole may interact with ER and bind to oestrogen response elements (EREs) in target tissues, forming a complex that acts as a transcription factor to initiate gene transcription (Hall and McDonnell 1999; Asiamah et al. 2021). This process can alter the expression of specific genes, thereby facilitating the biological effects of oestrogen in target cells. Thus, anethole may mimic the intracellular effects of oestrogen, potentially maintaining the continuity of oestrogen in the target cells. Moreover, the high oestrogen levels and gene expression associated with the reproductive axis in the FEO group in the current study supported this hypothesis.

Supplementing the diet with 350 mg/kg FEO improved the FCR of laying hens in both the early and late phases. This result is consistent with those of previous studies by Abou‐Elkhair, Selim, and Hussein (2018) and Samantaray and Nayak (2022). However, this outcome was contrary to that reported by Buğdaycı et al. (2018), who found no effect on FCR in laying quails fed fennel seeds. In addition, various studies have shown that peppermint oil improves the FCR, whereas Enviva essential oil and lavender essential oil do not affect FCR, and mint essential oil deteriorates it (Ding et al. 2017; Abdel‐Wareth and Lohakare 2020; Torki, Mohebbifar, and Mohammadi 2021). The beneficial effects of FEO on FCR can be explained by the findings of previous studies. First, it has been indicated that FEO works by reducing oxidative stress against harmful agents such as pathogens or toxic metabolites, thereby supporting animal health (Shahat et al. 2011). Second, FEO can activate endogenous digestive enzymes and other digestive secretions (such as bile and mucus), facilitating the more effective utilization of nutrients in the liver (Platel and Srinivasan 2001). Finally, FEO has been observed to expand the intestinal surface area, which is effective for nutrient absorption and positively influences the balance of the intestinal microbiota (İpçak, Alçiçek, and Denli 2024). Therefore, FEO may have improved the FCR by enhancing nutrient absorption and utilization through the mechanisms mentioned above.

Another important finding was that FEO supplementation, particularly the inclusion of 350 mg/kg FEO, increased egg weight and egg mass in the early laying phase. However, in the late laying phase, only egg mass was enhanced in hens fed 350 mg/kg FEO. In accordance with our findings, previous studies have demonstrated that fennel seed powder (Abou‐Elkhair, Selim, and Hussein 2018), peppermint oil (Abdel‐Wareth and Lohakare 2020), fennel oil (Samantaray and Nayak 2022) and lavender essential oil (Torki, Mohebbifar, and Mohammadi 2021) increase egg mass. However, Zhang et al. (2022) suggested that Shudi Erzi San did not affect egg weight, which matches our late‐laying phase finding. Nobakht and Mehman Navaz (2010) indicated that the inclusion of phytogenics in poultry diets leads to an increase in digestive enzymes and an improvement in the anatomical condition of the intestines. They stated that this directly contributes to an increase in egg weight by enhancing the absorption and storage of nutrients, including calcium, in a more effective and larger quantity. In this context, FEO may have promoted the secretion of digestive enzymes in chickens, potentially contributing to an increase in egg weight.

The current study found that FEO supplementation decreased feed intake during the early laying phase but did not affect feed intake during the late laying phase of hens. The feed intake result in the early laying phase was in agreement with those reported by Abou‐Elkhair, Selim, and Hussein (2018). Many earlier studies have indicated that diets supplemented with fennel seed fruit (Gharaghani, Shariatmadari, and Torshizi 2015), anise seed or oil (Beshara 2018), Enviva essential oil (Ding et al. 2017) and *Macleaya cordata* extract (Wang et al. 2022) had no effect on feed intake, which is in line with our results in late laying phase. Other studies have reported that peppermint oil (Abdel‐Wareth and Lohakare 2020) and fennel oil (Samantaray and Nayak 2022) increased feed intake, which is inconsistent with our findings. Indeed, the decrease in feed intake observed in our study could be attributed to the antimicrobial efficacy of FEO, which potentially eliminates competing pathogenic microorganisms, thereby reducing nutrient competition and allowing hens fed with FEO to utilize the provided feed more efficiently (İpçak, Alçiçek, and Denli 2024). Verstegen and Williams (2002) noted that approximately 6% of the net energy in pig diets may be lost owing to microbial consumption in the small intestine. Furthermore, supplementation of FEO in an encapsulated form to chickens could enhance the bioavailability of this active ingredient, thereby increasing its utilization efficiency. Balasubramanian, Park, and Kim (2016) reported that the encapsulation of additives, such as phytogenic substances or organic acids, can enhance their release in targeted areas, such as the small intestine, thereby potentially improving animal performance. Overall, the significant effects of FEO on laying performance can be attributed to improved feed utilization and subsequent improvements in hormonal balance, ovarian characteristics and overall health. Moreover, the inconsistency observed in previous studies could be attributed to the biological and pharmacological effects of the phytogenics or their active components. These effects encompass variables such as the proportion of diets, utilization and absorption, forms of feeding and diversity in the genotypes and ages of the hens.

Ovarian follicles are pivotal in reproductive biology and significantly influence egg production (Yoshimura and Barua 2017). They can be classified into two categories on the basis of their developmental stage: pre‐hierarchical and hierarchical (or pre‐ovulatory) follicles (Johnson 2015). Pre‐hierarchical follicles are further divided into SWFs, LWFs and SYFs. In contrast, hierarchical follicles consist of large yellow follicles (LYFs) and are ordered from largest to smallest as F1 through F5 or F6, according to their size and developmental progression (Onagbesan, Bruggeman, and Decuypere 2009). Following maturation and ovulation of the F1 follicle, the F2 and F3 follicles are sequentially promoted to the positions of the new F1 and F2, respectively, whereas LYF transitions to a pre‐ovulatory state (Johnson and Woods 2009; Asiamah et al. 2021). Several signalling pathways and regulatory mechanisms play crucial roles in follicular development. Interestingly, our study found that, despite numerical variations, there were no significant differences in either the number or weight of pre‐hierarchical and hierarchical follicles during the early and late laying phases of hens. Taherkhani, Ghiasi, and Ebrahimi (2018) observed that fennel seed extract (10 mg/kg) did not affect the number of LYFs and SWFs but increased SYFs and LWFs, which was somewhat consistent with our study. Long et al. (2017) also suggested that dietary supplementation with octacosanol increased the number of pre‐ovulatory follicles, SYFs and LWFs. In a recent study by Ma et al. (2024) demonstrated that in lean line hens, pre‐hierarchical follicles increased with age, whereas hierarchical follicles decreased across both lean and fat line hens. Our findings were consistent with the increase in pre‐hierarchical follicles reported by Ma et al. (2024) but did not align with the reported increase in hierarchical follicles. These observed differences might be attributed to the varying age groups and genetic lines of laying hens.

An increase in the number of cracked eggs is one of the most significant economic challenges in industrial poultry farming. Eggshell thickness is the most crucial factor influencing the rate of cracked eggs (Zhang et al. 2022). In the present study, a significant reduction in the rate of cracked eggs during the early laying stages was observed in chickens supplemented with 350 mg/kg FEO. However, only numerical improvements in the rate of cracked eggs were detected during the late laying stages. Xiao et al. (2022) observed that a combined essential oil decreased the egg‐breaking rate, which is in line with our early laying phase results. Moreover, various studies have shown that diets supplemented with Enviva essential oil (Ding et al. 2017), fennel seed extract (Taherkhani, Ghiasi, and Ebrahimi 2018) and peppermint oil (Abdel‐Wareth and Lohakare 2020) increase eggshell thickness. Other studies have indicated that diets supplemented with *Moringa oleifera* seed powder (Abou‐Elkhair et al. 2020), lavender and mint essential oils, their combination (Torki, Mohebbifar, and Mohammadi 2021), and Shudi Erzi San (Zhang et al. 2022) did not change eggshell thickness. Steroid hormones, particularly oestradiol, play critical roles in reproductive processes and egg production in laying hens and are crucial for the regulation of calcium metabolism through various mechanisms (Dojană et al. 2015). Bolscher et al. (1999) reported that oestradiol increased blood calcium levels by enhancing calcium absorption through the digestive system. Oestrogen facilitates the release of calcium ions necessary for the formation of eggshells from the bones (Yamada et al. 2021). This situation leads to an increase in blood calcium levels, which, on one hand, promotes the synthesis of 25‐dihydroxycholecalciferol (Yazarlou et al. 2012) and, on the other hand, supports the growth of the oviduct and the transportation of precursors such as proteins, fats and vitamins necessary for egg formation from the liver to the developing follicles (Nakada, Koja, and Tanaka 1994; Sah and Mishra 2018). Furthermore, Taherkhani, Ghiasi, and Ebrahimi (2018) reported that fennel extract activates hydroxylase, which increases calcium absorption through the digestive system, thereby facilitating the production of the active form of vitamin D3 and contributing to increased serum calcium levels. Our findings indicated that FEO enhances calcium absorption and storage by increasing serum oestrogen levels, thereby improving eggshell quality and reducing the rate of egg breakage.

In this study, diets supplemented with FEO increased serum *E*2, FSH and LH levels in both the early and late laying phases of the hens. Each hormone plays a specific role in various stages of follicular growth, maturation and ovulation in laying hens (Zhang et al. 2022). This process is initiated by the release of GnRH, which triggers the anterior pituitary gland to release of FSH and LH (Vézina, Salvante, and Williams 2003). FSH is essential for ovarian follicle growth and maturation, stimulates the production of oestrogen and influences the development of multiple follicles early in the egg‐laying cycle (Johnson and Woods 2009). LH triggers ovum release by causing the mature follicle to detach or rupture at the stigma site within the ovary (Oguike et al. 2005). FSH and LH are crucial for stimulating the secretion of oestrogen, progesterone and testosterone, which are key sex steroid hormones that regulate the ovulatory cycle in hens (Vézina, Salvante, and Williams 2003; Peng et al. 2024). Oestradiol (*E*2) plays a role in preparing the oviduct, initiating the next ovulatory cycle, forming the eggshell and supporting secondary sexual characteristics (Etches and Schoch 1984; Mishra, Sah, and Wasti 2019). Moreover, *E*2 levels increase as follicles mature, priming the reproductive tract for ovum formation, and *E*2 signalling not only regulates gonadotropin secretion (FSH and LH) but also modulates the functions of gonadotropins within the ovary (Richards, 1980; Lee et al. 2021). Progesterone, secreted by the largest follicles in the later stages of follicle development near ovulation, helps regulate the interval between ovulations in conjunction with *E*2 and promotes the onset of the LH surge for the release of the yolk (ovum) (Johnson 2015). Ovulation was induced at the peak LH levels. Following ovulation, as progesterone levels diminish, the function and influence of *E*2 persist. *E*2 then positively stimulates the hypothalamus and subsequently the pituitary gland, thereby assisting in the regulatory release of FSH and LH, which are essential for restarting a new ovulatory cycle (Asiamah et al. 2021). Our findings also indicate that FEO significantly sustains serum *E*2 levels during the late laying phase, thereby positively affecting egg production, supporting our hypothesis. These results are consistent with those of Long et al. (2017), who observed that dietary supplementation with octacosanol leads to increased levels of serum *E*2, FSH and LH. Similarly, Saleh, Ahmed, and Ebeid (2019) found that flaxseeds, fenugreek seeds and their combination as sources of phytoestrogens significantly enhanced the plasma levels of LH, FSH and *E*2. Additionally, *M. cordata* extract increased serum FSH levels and decreased LH levels in laying hens (Wang et al. 2022).

Moreover, in the current study, FEO supplementation also increased progesterone and testosterone levels in the bodies of hens during the early and late laying phases. Progesterone, along with oestrogen, is integral in managing the timing of ovulation, which stabilizes the egg‐laying cycle and is crucial for maximizing efficiency and productivity in commercial egg‐laying farming by guaranteeing that hens lay eggs at a consistent and optimal rate (Zhu et al. 2019). The increase in progesterone levels observed in our study can be attributed to the interaction between anethole and ER, thereby activating oestrogen‐dependent progesterone receptors (Haslam 1988; Çetin et al. 2022). Furthermore, testosterone, which is primarily linked to male reproductive functions, influences female chicken behaviour in mating and egg production. Even at lower levels, testosterone contributes to regulating aggression and may play a role in pre‐ovulatory follicle maturation by stimulating granulosa progesterone production (Rangel et al. 2009; Goerlich, Dijkstra, and Groothuis 2010). Rangel et al. (2009) also reported that testosterone stimulates progesterone production in granulosa cells of hen pre‐ovulatory hierarchical follicles irrespective of the maturational state, acting alone or in combination with LH. Furthermore, they proposed that testosterone promotes granulosa cell maturation to facilitate the pre‐ovulatory release of LH. Our findings suggest that FEO is an oestrogen mediator that significantly stimulates hormone secretion, resulting in maintenance of the ovulatory cycle, thereby increasing egg production. Additionally, an increase in oestrogen synthesis could lead to enhanced development of the oviduct and follicles, directly improving the egg weight and shell quality.

To explore the possible molecular mechanisms by which FEO improves the performance of laying hens, we examined gene expression levels in blood and ovary tissues. Understanding differences in gene expression related to follicular development in hens can provide critical insights into the management of nutritional conditions to enhance egg production in poultry farming (Du et al. 2020). We found that the expression levels of the follicle development‐associated genes FSHR and ERβ (ESR2) in both the blood and ovarian tissues from the FEO supplementation groups were significantly increased relative to those from the control group during both the early and late laying phases. Relatedly, ESR2 expression levels were higher in late‐laying hens than in early laying hens. Drummond and Fuller (2009) noted that ERβ is the predominant oestrogen receptor in the ovary, with the adult ovary being the site associated with the highest level of ERβ expression. In previous studies conducted on laying hens, the extract of *M. cordata* was shown to increase the expression of ESR2 and FSHR in the blood (Wang et al. 2022), whereas dietary octacosanol was found to enhance the expression of FSHR and LH receptor (LHR) in pre‐ovulatory granulosa cells (Long et al. 2017), which is consistent with our results. Additionally, Abou‐Elkhair et al. (2020) reported that 0.3% *M. oleifera* seed powder increased the expression of ESR2 and FSHR in the ovaries of laying quails.

Furthermore, the elevated levels of oestrogen hormone, particularly in its *E*2 form, secreted from the ovaries prior to ovulation, target kisspeptin (KP) neurons and GnRH neurons in the hypothalamus, triggering the regulation of expression and release of these neurons (Lee et al. 2021). In this mechanism, the roles of the ER ERα (ESR1) and ERβ, which are located on the cell surface or within the cell and possess a specific ability to bind to the oestrogen hormone, are crucial (Chen et al. 2019). These receptors are expressed in KP neurons, GnRH neurons in the hypothalamus and in pituitary gonadotrophs (Harter, Kavanagh, and Smith 2018). ERs mediate the cellular effects of oestrogen and facilitate the interaction between the hormone and its target cells; however, the biological and physiological effects of oestrogen in the body vary depending on the presence and status of these receptors (Fuentes and Silveyra 2019). Specifically, ERβ plays a pivotal role in regulating GnRH and gonadotropin secretion, thereby determining gonadotropin levels and function during ovulation (Novaira et al. 2018). ERβ is essential for ovulation. Therefore, a potential decrease in serum *E*2 could be associated with a deficiency in ERβ, leading to a weakened pre‐ovulatory gonadotropin surge and, consequently, a decrease in egg production (Jayes et al. 2014). In this context, on the basis of the oestrogenic properties of FEO, it can be stated that the increase in ER not only enhances the efficacy of oestrogen in the body but also leads to an increase in the levels of FSH and LH in the serum, thereby consistently supporting the ovulatory cycle. STAR plays a critical role in steroidogenesis (Rangel et al. 2009). Steroidogenesis is the biosynthesis of sex steroid hormones (specifically, oestrogen, progesterone and sometimes testosterone). This process involves transportation of cholesterol, the building block of steroid hormones, into the mitochondria, where it is first converted to pregnenolone and then transformed into steroid hormones via various enzymatic pathways (Hales et al. 2005). Particularly in the ovaries and, to a lesser extent, in the adrenal glands, this process cannot occur without the assistance of the STAR protein (Harvey and Everett 2003). In this context, on the basis of the findings of our study, it is likely that the increased expression of STAR in chickens fed FEO supplementation triggered the formation of the STAR protein, which in turn could have contributed to the elevation of serum levels of oestrogen and progesterone. A previous study reported that a diet supplemented with *M. oleifera* seed powder increased the expression of STAR in the ovaries of laying quails, which was consistent with our results (Abou‐Elkhair et al. 2020).

Additionally, in this study, PRLR expression was examined because of its galactogenic properties, which are attributed to the structural similarity between anethole and dopamine (Badgujar, Patel, and Bandivdekar 2014). Prolactin is a hormone that plays a role in milk production in mammals. However, it also plays a crucial role in initiating and maintaining brooding behaviour in chickens (Cui et al. 2006; Mo et al. 2022). High prolactin levels are associated with the onset of brooding behaviour, and physiological changes occurring in the body of hens during this period can temporarily halt follicular development and ovulation (Raju et al. 2013). This can lead to a decrease in egg production. Indeed, our findings indicated that, especially in the early laying phase, supplementation with 350 mg/kg FEO increased PRLR mRNA expression. However, this increase remained at the transcriptional level and did not negatively affect egg production due to the lack of reflection at the protein level through post‐translational mechanisms. This effect could also be supported by the absence of changes in the serum prolactin levels. Moreover, a positive correlation between FSHR, ESR2 and STAR expression levels and egg production, along with a negative correlation between PRLR expression levels and egg production, confirmed the positive effects of FEO on laying performance. This result is in agreement with that obtained by Abou‐Elkhair et al. (2020), who reported that *M. oleifera* seed powder increased PRLR expression in the ovaries of laying quails. In general, Lee et al. (2021) reported that ERs, classical ligand‐activated transcriptional regulators, play crucial roles in the regulation of gonadotropin secretion from the HPG axis, as well as gonadotropin function in target organs. Consequently, it can be said that anethole interacts with ER, inducing the mRNA expression of genes related to the reproductive axis, leading to an increase in serum levels of hormones.

## 5 Conclusion

In conclusion, dietary encapsulated FEO supplementation enhanced hen‐day egg production, accompanied by increased serum oestradiol, FSH, LH and progesterone concentrations, and induced both blood and ovarian mRNA expression of the major reproductive axis‐related genes ESR2 and FSHR, which are involved in follicle growth and ovulation. Supplementation with 350 mg/kg encapsulated FEO was more effective in improving laying performance, stimulating reproductive hormones and modulating related gene expression through the synergy of molecular and endocrine pathways. These results suggest that FEO is a promising phytoestrogen that can be used in the diets of laying hens. However, future research should focus on thoroughly understanding the complex effects of phytoestrogens on the body and determining their biosafety.

## Author Contributions

H.H.I. contributed to conceptualization, investigation, data collection, data analysis, writing and the preparation of the original draft. M.D. oversaw project administration, contributed to conceptualization and was involved in editing. B.Y. and S.B. provided supervision and contributed to data analysis.

## Ethics Statement

The authors confirm that the ethical policies of the journal, as noted on the journal's author guidelines page, have been adhered to, and appropriate ethical review committee approval has been received. The authors also confirm that they followed the guidelines of the US National Research Council for the Care and Use of Laboratory Animals. The Experimental Animal Ethics Committee of the Dicle University (Protocol number: 2020/07) approved all procedures involving animals.

## Conflicts of Interest

The authors declare no conflicts of interest.

### Peer Review

The peer review history for this article is available at https://publons.com/publon/10.1002/vms3.70150.

## Data Availability

All data are reported in the article.

## References

[vms370150-bib-0001] Abdel‐Wareth, A. A. A. , and J. D. Lohakare . 2014. “Effect of Dietary Supplementation of Peppermint on Performance, Egg Quality, and Serum Metabolic Profile of Hy‐Line Brown Hens During the Late Laying Period.” Animal Feed Science and Technology 197: 114–120. 10.1016/j.anifeedsci.2014.07.007.

[vms370150-bib-0002] Abdel‐Wareth, A. A. A. , and J. D. Lohakare . 2020. “Productive Performance, Egg Quality, Nutrients Digestibility, and Physiological Response of Bovans Brown Hens Fed Various Dietary Inclusion Levels of Peppermint Oil.” Animal Feed Science and Technology 267: 114554. 10.1016/j.anifeedsci.2020.114554.

[vms370150-bib-0003] Abou‐Elkhair, R. , H. Abdo Basha , W. Slouma Hamouda Abd El Naby , et al. 2020. “Effect of a Diet Supplemented With the *Moringa oleifera* Seed Powder on the Performance, Egg Quality, and Gene Expression in Japanese Laying Quail Under Heat‐Stress.” Animals 10, no. 5: 809. 10.3390/ani10050809. 32392810 PMC7278701

[vms370150-bib-0004] Abou‐Elkhair, R. , S. Selim , and E. Hussein . 2018. “Effect of Supplementing Layer Hen Diet With Phytogenic Feed Additives on Laying Performance, Egg Quality, Egg Lipid Peroxidation and Blood Biochemical Constituents.” Animal Nutrition 4, no. 4: 394–400. 10.1016/j.aninu.2018.05.009. 30564759 PMC6286622

[vms370150-bib-0005] AOAC International . 2000. Official Methods of Analysis. 17th Edition, The Association of Official Analytical Chemists: Gaithersburg, MD, USA.

[vms370150-bib-0006] Asiamah, C. A. , Y. Liu , R. Ye , et al. 2021. “Immunohistochemistry and Expression Profile of Estrogen Receptor 2 Gene in Different Grade Size Ovarian Follicles of Leizhou Black Ducks.” Research Square. https://assets.researchsquare.com/files/rs‐839094/v1_covered.pdf?c=1631878904.

[vms370150-bib-0007] Badgujar, S. B. , V. V. Patel , and A. H. Bandivdekar . 2014. “ *Foeniculum vulgare* Mill: A Review of Its Botany, Phytochemistry, Pharmacology, Contemporary Application, and Toxicology.” BioMed Research International 2014: 1–32. 10.1155/2014/842674. PMC4137549 25162032

[vms370150-bib-0008] Balasubramanian, B. , J. W. Park , and I. H. Kim . 2016. “Evaluation of the Effectiveness of Supplementing Micro‐Encapsulated Organic Acids and Essential Oils in Diets for Sows and Suckling Piglets.” Italian Journal of Animal Science 15, no. 4: 626–633. 10.1080/1828051X.2016.1222243.

[vms370150-bib-0009] Beshara, M. M. 2018. “Effect of Dietary Cholecalciferol and Anethole Source on Productive and Reproductive Performance of Local Laying Hens 1‐From 25 to 40 Weeks of Age.” Egyptian Poultry Science Journal 38, no. 4: 1025–1046. 10.21608/EPSJ.2018.22688.

[vms370150-bib-0010] Bolscher, M. , J. C. Netelenbos , R. Barto , L. M. van Buuren , and W. J. van der vijgh . 1999. “Estrogen Regulation of Intestinal Calcium Absorption in the Intact and Ovariectomized Adult Rat.” Journal of Bone and Mineral Research 14, no. 7: 1197–1202. 10.1359/jbmr.1999.14.7.1197. 10404021

[vms370150-bib-0011] Brouklogiannis, I. P. , E. C. Anagnostopoulos , E. Griela , V. V. Paraskeuas , and K. C. Mountzouris . 2023. “Dietary Phytogenic Inclusion Level Affects Production Performance and Expression of Ovarian Cytoprotective Genes in Laying Hens.” Poultry Science 102, no. 4: 102508. 10.1016/j.psj.2023.102508. PMC9932107 36739797

[vms370150-bib-0012] Buğdaycı, K. E. , F. K. Oğuz , M. N. Oğuz , and E. Kuter . 2018. “Effects of Fennel Seed Supplementation of Ration on Performance, Egg Quality, Serum Cholesterol, and Total Phenol Content of Egg Yolk of Laying Quails.” Revista Brasileira De Zootecnia 47: e20170160. 10.1590/rbz4720170160.

[vms370150-bib-0013] Çetin, N. C. , İ. Erdem , Ö. F. Durusoy , S. Alaşahan , and T. Çimrin . 2022. “The Effect of Supplementation of Fennel (*Foeniculum vulgare* Mill.) to the Feed on Egg Production, Slaughter and Carcass Characteristics, Formation of Parasites in the Intestine and Spermatological Quality in Japanese Quail During the Laying Period.” Dicle University Journal of Faculty of Veterinary Medicine 15, no. 2: 85–92. 10.47027/duvetfd.1159507.

[vms370150-bib-0014] Chen, C. , X. Gong , X. Yang , et al. 2019. “The Roles of Estrogen and Estrogen Receptors in Gastrointestinal Disease.” Oncology Letters 18, no. 6: 5673–5680. 10.3892/ol.2019.10983. 31788039 PMC6865762

[vms370150-bib-0015] Christaki, E. V. , E. M. Bonos , and P. C. Florou‐Paneri . 2011. “Use of Anise Seed and/or α‐Tocopheryl Acetate in Laying Japanese Quail Diets.” South African Journal of Animal Science 41, no. 2: 126–133. https://journals.co.za/doi/abs/10.10520/EJC94798.

[vms370150-bib-0016] Cui, J. X. , H. L. Du , Y. Liang , X. M. Deng , N. Li , and X. Q. Zhang . 2006. “Association of Polymorphisms in the Promoter Region of Chicken Prolactin With Egg Production.” Poultry Science 85, no. 1: 26–31. 10.1093/ps/85.1.26. 16493942

[vms370150-bib-0017] Ding, X. , Y. Yu , Z. Su , and K. Zhang . 2017. “Effects of Essential Oils on Performance, Egg Quality, Nutrient Digestibility and Yolk Fatty Acid Profile in Laying Hens.” Animal Nutrition 3, no. 2: 127–131. 10.1016/j.aninu.2017.03.005. 29767138 PMC5941116

[vms370150-bib-0018] Dojană, N. , G. Cotor , I. Codreanu , and R. Bălăceanu . 2015. “The Effect of Experimental 17‐Beta Estradiol Administering on Calcium Metabolism Regulation in Young Laying Hens.” Bulletin UASVM Veterinary Medicine 72, no. 1: 90–92. 10.15835/buasvmcn-vm:10854.

[vms370150-bib-0019] Drummond, A. E. , and P. J. Fuller . 2009. “The Importance of ERbeta Signalling in the Ovary.” Journal of Endocrinology 205, no. 1: 15–23. 10.1677/joe-09-0379. 20019181

[vms370150-bib-0020] Du, Y. , L. Liu , Y. He , T. Dou , J. Jia , and C. J. B. P. S. Ge . 2020. “Endocrine and Genetic Factors Affecting Egg Laying Performance in Chickens: A Review.” British Poultry Science 61, no. 5: 538–549. 10.1080/00071668.2020.1758299. 32306752

[vms370150-bib-0021] Etches, R. J. , and J. P. Schoch . 1984. “A Mathematical Representation of the Ovulatory Cycle of the Domestic Hen.” British Poultry Science 25, no. 1: 65–76. 10.1080/13632758408454843. 6713234

[vms370150-bib-0022] Food and Drug Administration . 2018. Bioanalytical Method Validation: Guidance for Industry. Silver Spring, MD: FDA. Accessed January 2024. https://www.fda.gov/files/drugs/published/Bioanalytical‐Method‐Validation‐Guidance‐for‐Industry.pdf.

[vms370150-bib-0080] Fuentes, N. , and P. Silveyra . 2019. “Estrogen Receptor Signaling Mechanisms.” Advances in Protein Chemistry and Structural Biology, 116: 135–170. 10.1016/bs.apcsb.2019.01.001. 31036290 PMC6533072

[vms370150-bib-0023] Gharaghani, H. , F. Shariatmadari , and M. A. Torshizi . 2015. “Effect of Fennel (*Foeniculum vulgare* Mill.) Used as a Feed Additive on the Egg Quality of Laying Hens Under Heat Stress.” Brazilian Journal of Poultry Science 17: 199–207. 10.1590/1516-635X1702199-208.

[vms370150-bib-0024] Ghasemian, A. , A. H. Al‐Marzoqi , S. K. S. Mostafavi , Y. K. Alghanimi , and M. Teimouri . 2020. “Chemical Composition and Antimicrobial and Cytotoxic Activities of *Foeniculum vulgare* Mill Essential Oils.” Journal of Gastrointestinal Cancer 51: 260–266. 10.1007/s12029-019-00241-w. 31069662

[vms370150-bib-0025] Goerlich, V. C. , C. Dijkstra , and T. G. Groothuis . 2010. “Effects of In Vivo Testosterone Manipulation on Ovarian Morphology, Follicular Development, and Follicle Yolk Testosterone in the Homing Pigeon.” Journal of Experimental Zoology Part A: Ecological Genetics and Physiology 313, no. 6: 328–338. 10.1002/jez.600. 20336796

[vms370150-bib-0026] Gouin, S. 2004. “Microencapsulation: Industrial Appraisal of Existing Technologies and Trends.” Trends in Food Science & Technology 15, no. 7–8: 330–347. 10.1016/j.tifs.2003.10.005.

[vms370150-bib-0027] Griffin, C. , G. Flouriot , V. Sonntag‐Buck , and F. Gannon . 1999. “Two Functionally Different Protein Isoforms Are Produced From the Chicken Estrogen Receptor‐α Gene.” Molecular Endocrinology 13, no. 9: 1571–1587. 10.1210/mend.13.9.0336. 10478847

[vms370150-bib-0028] Haglan, M. M. , and A. A. Majed . 2022. “Effect of Dıetary Supplementatıon of Fennel Seeds (*Foenıculum vulgare*) on Improvıng Egg Qualıty in Layıng Hens.” Sciences 20, no. 2: 377–390. 10.32649/ajans.2022.176586.

[vms370150-bib-0029] Hales, D. B. , J. A. Allen , T. Shankara , et al. 2005. “Mitochondrial Function in Leydig Cell Steroidogenesis.” Annals of the New York Academy of Sciences 1061, no. 1: 120–134. 10.1196/annals.1336.014. 16469751

[vms370150-bib-0030] Hall, J. M. , and D. P. McDonnell . 1999. “The Estrogen Receptor β‐Isoform (ERβ) of the Human Estrogen Receptor Modulates ERα Transcriptional Activity and Is a Key Regulator of the Cellular Response to Estrogens and Antiestrogens.” Endocrinology 140, no. 12: 5566–5578. 10.1210/endo.140.12.7179. 10579320

[vms370150-bib-0031] Harter, C. J. , G. S. Kavanagh , and J. T. Smith . 2018. “The Role of Kisspeptin Neurons in Reproduction and Metabolism.” Journal of Endocrinology 238, no. 3: R173–R183. 10.1530/JOE-18-0108. 30042117

[vms370150-bib-0032] Harvey, P. W. , and D. J. Everett . 2003. “The Adrenal Cortex and Steroidogenesis as Cellular and Molecular Targets for Toxicity: Critical Omissions From Regulatory Endocrine Disrupter Screening Strategies for Human Health?” Journal of Applied Toxicology: An International Journal 23, no. 2: 81–87. 10.1002/jat.896. 12666151

[vms370150-bib-0033] Haslam, S. Z. 1988. “Cell to Cell Interactions and Normal Mammary Gland Function.” Journal of Dairy Science 71, no. 10: 2843–2854. 10.3168/jds.S0022-0302(88)79880-X. 3204193

[vms370150-bib-0034] Hocking, P. M. 1996. “Role of Body Weight and Food Intake After Photostimulation on Ovarian Function at First Egg in Broiler Breeder Females.” British Poultry Science 37, no. 4: 841–851. 10.1080/00071669608417913. 8894228

[vms370150-bib-0035] İpçak, H. H. , A. Alçiçek , and M. Denli . 2024. “Dietary Encapsulated Fennel Seed (*Foeniculum vulgare* Mill.) Essential Oil Supplementation Improves Performance, Modifies the Intestinal Microflora, Morphology, and Transcriptome Profile of Broiler Chickens.” Journal of Animal Science 102: 1–20. 10.1093/jas/skae035. PMC10943331 38330242

[vms370150-bib-0036] Jayes, F. L. , K. A. Burns , K. F. Rodriguez , G. E. Kissling , and K. S. Korach . 2014. “The Naturally Occurring Luteinizing Hormone Surge Is Diminished in Mice Lacking Estrogen Receptor Beta in the Ovary.” Biology of Reproduction 90, no. 2: 21–24. 10.1095/biolreprod.113.113316. PMC4076404 24337314

[vms370150-bib-0037] Johnson, A. L. 2014. “The Avian Ovary and Follicle Development: Some Comparative and Practical Insights.” Turkish Journal of Veterinary & Animal Sciences 38, no. 6: 660–669. 10.3906/vet-1405-6.

[vms370150-bib-0038] Johnson, A. L. 2015. “Reproduction in the Female.” In Sturkie's Avian Physiology, 635–665. Cambridge, MA: Academic Press. 10.1016/B978-0-12-407160-5.00028-2.

[vms370150-bib-0039] Johnson, A. L. , and D. C. Woods . 2009. “Dynamics of Avian Ovarian Follicle Development: Cellular Mechanisms of Granulosa Cell Differentiation.” General and Comparative Endocrinology 163, no. 1–2: 12–17. 10.1016/j.ygcen.2008.11.012. 19059411

[vms370150-bib-0040] Lee, C. , J. Kim , S. G. Shin , and S. Hwang . 2006. “Absolute and Relative QPCR Quantification of Plasmid Copy Number in *Escherichia coli* .” Journal of Biotechnology 123, no. 3: 273–280. 10.1016/j.jbiotec.2005.11.014. 16388869

[vms370150-bib-0041] Lee, E. B. , V. P. Chakravarthi , M. W. Wolfe , and M. K. Rumi . 2021. “ERβ Regulation of Gonadotropin Responses During Folliculogenesis.” International Journal of Molecular Sciences 22, no. 19: 10348. 10.3390/ijms221910348. 34638689 PMC8508937

[vms370150-bib-0042] Liu, L. , Z. Cui , Q. Xiao , et al. 2018. “Polymorphisms in the Chicken Growth Differentiation Factor 9 Gene Associated With Reproductive Traits.” BioMed Research International 2018: 9345473. 10.1155/2018/9345473. 30327782 PMC6169235

[vms370150-bib-0043] Long, L. , S. G. Wu , F. Yuan , H. J. Zhang , J. Wang , and G. H. Qi . 2017. “Effects of Dietary Octacosanol Supplementation on Laying Performance, Egg Quality, Serum Hormone Levels, and Expression of Genes Related to the Reproductive Axis in Laying Hens.” Poultry Science 96, no. 4: 894–903. 10.3382/ps/pew316. 27665009

[vms370150-bib-0044] Ma, Y. , B. Cheng , S. Zhou , et al. 2024. “Comparative Analyses of Laying Performance and Follicular Development Characteristics Between Fat and Lean Broiler Lines.” Poultry Science 103, no. 1: 103250. 10.1016/j.psj.2023.103250. PMC10667750 37992620

[vms370150-bib-0045] Mishra, B. , N. Sah , and S. Wasti . 2019. “Genetic and Hormonal Regulation of Egg Formation in the Oviduct of Laying hens.” In Poultry—An Advanced Learning, 1–10. London: IntechOpen. 10.5772/intechopen.85011.

[vms370150-bib-0046] Mo, G. , B. Hu , P. Wei , Q. Luo , and X. Zhang . 2022. “The Role of Chicken Prolactin, Growth Hormone and Their Receptors in the Immune System.” Frontiers in Microbiology 13: 900041. 10.3389/fmicb.2022.900041. 35910654 PMC9331192

[vms370150-bib-0047] Nakada, T. , Z. Koja , and K. Tanaka . 1994. “Effect of Progesterone on Ovulation in the Hypophysectomised Hen.” British Poultry Science 35, no. 1: 153–156. 10.1080/00071669408417680. 8199885

[vms370150-bib-0048] National Research Council (NRC) . 1994. Nutrient Requirements of Poultry. Washington, DC: National Academy Press.

[vms370150-bib-0049] Naz, R. K. 2004. Endocrine Disruptors: Effects on Male and Female Reproductive Systems. 2nd Edition, Boca Raton, FL, USA: CRC Press.

[vms370150-bib-0081] Nobakht, A. , and Y. Mehman Navaz . 2010. “Investigation the Effects of Using of Zizaphora (*Thymyus valgaris*), Peppermint (*Lamiaceae menthapiperita*), Menta Pulagum (*Oreganum valgare*) Medical Plants on Performance, Egg Quality, Blood and Immunity Parameters of Laying Hens.” Iranian Journal of Animal Science 41, no: 2. Accessed June 2023. https://ijas.ut.ac.ir/article_21503_en.html?lang=fa.

[vms370150-bib-0050] Novaira, H. J. , A. L. Negron , J. B. Graceli , et al. 2018. “Impairments in the Reproductive Axis of Female Mice Lacking Estrogen Receptor β in GnRH Neurons.” American Journal of Physiology‐Endocrinology and Metabolism 315, no. 5: E1019–E1033. 10.1152/ajpendo.00173.2018. 30040478 PMC6293171

[vms370150-bib-0051] Oguike, M. A. , G. Igboeli , S. N. Ibe , and M. O. Ironkwe . 2005. “Physiological and Endocrinological Mechanisms Associated With Ovulatory Cycle and Induced‐Moulting in the Domestic Chicken—A Review.” World's Poultry Science Journal 61, no. 4: 625–632. 10.1079/WPS200574.

[vms370150-bib-0052] Onagbesan, O. , V. Bruggeman , and E. Decuypere . 2009. “Intra‐Ovarian Growth Factors Regulating Ovarian Function in Avian Species: A Review.” Animal Reproduction Science 111, no. 2–4: 121–140. 10.1016/j.anireprosci.2008.09.017. 19028031

[vms370150-bib-0053] Peng, J. , W. Huang , M. Yang , et al. 2024. “Characteristics of Glucolipid Metabolism and Oxidative Stress in Breeding Pigeons (*Columba livia*) During Lactation.” Journal of Animal Physiology and Animal Nutrition 108, no. 1: 148–162. 10.1111/jpn.13875. 37609936

[vms370150-bib-0054] Platel, K. , and K. Srinivasan . 2001. “Studies on the Influence of Dietary Spices on Food Transit Time in Experimental Rats.” Nutrition Research 21, no. 9: 1309–1314. 10.1016/S0271-5317(01)00331-1.

[vms370150-bib-0055] Raju, G. A. R. , R. Chavan , M. Deenadayal , et al. 2013. “Luteinizing Hormone and Follicle Stimulating Hormone Synergy: A Review of Role in Controlled Ovarian Hyper‐Stimulation.” Journal of Human Reproductive Sciences 6, no. 4: 227–234. 10.4103/0974-1208.126285. 24672160 PMC3963304

[vms370150-bib-0056] Rangel, P. L. , A. Rodríguez , S. Rojas , P. J. Sharp , and C. G. Gutierrez . 2009. “Testosterone Stimulates Progesterone Production and STAR, P450 Cholesterol Side‐Chain Cleavage and LH Receptor mRNAs Expression in Hen (*Gallus domesticus*) Granulosa Cells.” Reproduction (Cambridge, England) 138, no. 6: 961–969. 10.1530/REP-09-0071. 19710202

[vms370150-bib-0057] Reineccius, G. A. 1991. “Carbohydrates for Favor Encapsulation.” Food Technology 45: 144–147.

[vms370150-bib-0058] Richards, J. S. 1980. “Maturation of Ovarian Follicles: Actions and Interactions of Pituitary and Ovarian Hormones on Follicular Cell Differentiation.” Physiological Reviews 60, no. 1: 51–89. 10.1152/physrev.1980.60.1.51. 6243782

[vms370150-bib-0059] Sah, N. , and B. Mishra . 2018. “Regulation of Egg Formation in the Oviduct of Laying Hen.” World's Poultry Science Journal 74, no. 3: 509–522. 10.1017/S0043933918000442.

[vms370150-bib-0060] Saleh, A. A. , E. A. Ahmed , and T. A. Ebeid . 2019. “The Impact of Phytoestrogen Source Supplementation on Reproductive Performance, Plasma Profile, Yolk Fatty Acids and Antioxidative Status in Aged Laying Hens.” Reproduction in Domestic Animals 54, no. 6: 846–854. 10.1111/rda.13432. 30916364

[vms370150-bib-0061] Samantaray, L. , and Y. Nayak . 2022. “The Influences of Black Pepper, Turmeric and Fennel Essential Oils Supplementation in Feed on Egg Quality Characteristics of Layers.” Journal of Animal Health and Production 10, no. 4: 522–528. 10.17582/journal.jahp/2022/10.4.522.528.

[vms370150-bib-0062] Shahat, A. A. , A. Y. Ibrahim , S. F. Hendawy , et al. 2011. “Chemical Composition, Antimicrobial and Antioxidant Activities of Essential Oils From Organically Cultivated Fennel Cultivars.” Molecules (Basel, Switzerland) 16, no. 2: 1366–1377. 10.3390/molecules16021366. 21285921 PMC6259638

[vms370150-bib-0063] SPSS . 2013. PASW Statistics for Windows, v. 22.0, Statistical Package for the Social Sciences. Chicago: SPSS Inc.

[vms370150-bib-0064] TAEM . 2015. “Republic of Türkiye Ministry of Agriculture and Forestry Poultry Research Institute.” ATAK‐S Commercial Brown Layer Performance Features (*in Turkish*). Accessed January 2024. Available online: https://arastirma.tarimorman.gov.tr/tavukculuk/Belgeler/Hibrit%20Katalog%202015/Hibrit%20Katalog%202015%203lu.pdf.

[vms370150-bib-0065] Taherkhani, R. , H. Ghiasi , and M. Ebrahimi . 2018. “The Effect of Using Fennel on Plasma Estrogen and Performance of Laying Hens.” Journal of Biochemical Technology 2: 115–122.

[vms370150-bib-0066] Torki, M. , A. Mohebbifar , and H. Mohammadi . 2021. “Effects of Supplementing Hen Diet With *Lavandula angustifolia* and/or *Mentha spicata* Essential Oils on Production Performance, Egg Quality and Blood Variables of Laying Hens.” Veterinary Medicine and Science 7, no. 1: 184–193. 10.1002/vms3.343. 32864892 PMC7840192

[vms370150-bib-0067] TSE . 1997. “Turkish Standards Institution, Animal Feed—Determination of Sugar‐Luff‐Schoorl Method.” TSE. Accessed May 2023 Available online: https://www.gafta.com/write/MediaUploads/Contracts/2018/METHOD_10.1_SUGAR_‐_LUFF_SCHOORL_METHOD.pdf.

[vms370150-bib-0068] TSE . 2004. “Turkish Standards Institution, Animal Feed—Determination of Starch Content‐Polarimetric Method.” TSE. Accessed May 2023. Available online: https://cdn.standards.iteh.ai/samples/12866/06fffe8e565e4969808a82b973bea718/ISO‐6493‐2000.pdf.

[vms370150-bib-0069] Verstegen, M. W. , and B. A. Williams . 2002. “Alternatives to the Use of Antibiotics as Growth Promoters for Monogastric Animals.” Animal Biotechnology 13, no. 1: 113–127. 10.1081/ABIO-120005774. 12212936

[vms370150-bib-0070] Vézina, F. , K. G. Salvante , and T. D. Williams . 2003. “The Metabolic Cost of Avian Egg Formation: Possible Impact of Yolk Precursor Production?” Journal of Experimental Biology 206, no. 24: 4443–4451. 10.1242/jeb.00702. 14610029

[vms370150-bib-0071] Wang, F. , P. Zou , S. Xu , et al. 2022. “Dietary Supplementation of *Macleaya cordata* Extract and *bacillus* in Combination Improve Laying Performance by Regulating Reproductive Hormones, Intestinal Microbiota and Barrier Function of Laying Hens.” Journal of Animal Science and Biotechnology 13, no. 1: 118. 10.1186/s40104-022-00766-4. 36224643 PMC9559840

[vms370150-bib-0072] Xiao, G. , L. Zheng , X. Yan , et al. 2022. “Effects of Dietary Essential Oils Supplementation on Egg Quality, Biochemical Parameters, and Gut Microbiota of Late‐Laying Hens.” Animals 12, no. 19: 2561. 10.3390/ani12192561. 36230302 PMC9558990

[vms370150-bib-0073] Yamada, M. , C. Chen , T. Sugiyama , and W. K. Kim . 2021. “Effect of Age on Bone Structure Parameters in Laying Hens.” Animals 11, no. 2: 570. 10.3390/ani11020570. 33671735 PMC7926946

[vms370150-bib-0074] Yazarlou, M. , S. Sharifi , M. Melaki , M. Zahedi , and K. Bahmani . 2012. “The Effect of Fennel Seed Levels on the Physical and Qualitative Properties of Japanese Quail's Egg.” In The First National Seminar on the Management of Raising Poultry and Domestic Animals in the Tropical Regions, 948. Kerman, Branch: Shahid Bahonar University. 10.32649/ajas.2022.176586.

[vms370150-bib-0075] Yoshimura, Y. , and A. Barua . 2017. “Female Reproductive System and Immunology.” Advances in Experimental Medicine and Biology 1001: 33–57. 10.1007/978-981-10-3975-1_3. 28980228

[vms370150-bib-0076] Zhang, Y. , J. Meng , L. Zhang , et al. 2022. “Shudi Erzi San Relieves Ovary Aging in Laying Hens.” Poultry Science 101, no. 9: 102033. 10.1016/j.psj.2022.102033. PMC9356177 35926353

[vms370150-bib-0077] Zhu, G. , C. Fang , J. Li , C. Mo , Y. Wang , and J. Li . 2019. “Transcriptomic Diversification of Granulosa Cells During Follicular Development in Chicken.” Scientific Reports 9, no. 1: 5462. 10.1038/s41598-019-41132-1. 30940861 PMC6445143

